# Associations between three *XRCC1* polymorphisms and hepatocellular carcinoma risk: A meta-analysis of case-control studies

**DOI:** 10.1371/journal.pone.0206853

**Published:** 2018-11-08

**Authors:** Yao Xiong, Qian Zhang, Jiaxiang Ye, Shan Pan, Lianying Ge

**Affiliations:** 1 Department of Medical Oncology, The Affiliated Tumor Hospital of Guangxi Medical University, Nanning, Guangxi Zhuang Autonomous Region, China; 2 Department of Gynecologic Oncology, The Affiliated Tumor Hospital of Guangxi Medical University, Nanning, Guangxi Zhuang Autonomous Region, China; Duke Cancer Institute, UNITED STATES

## Abstract

Conflicting results have been obtained regarding the association between X-ray repair cross complementation group 1 (XRCC1) and susceptibility to hepatocellular carcinoma (HCC). In this study, associations between HCC and three polymorphisms (Arg194Trp, Arg280His, and Arg399Gln) were evaluated using a meta-analysis approach. PubMed, Web of Science, Cochrane Library, the Chinese National Knowledge Infrastructure, and the Wanfang standard database were systematically searched to identify all relevant case-control studies published through March 2018. A total of 32 case-control studies, including 13 that evaluated Arg194Trp, 14 that evaluated Arg280His, and 26 that evaluated Arg399Gln, were analyzed. In the entire study population, XRCC1 Arg399Gln was significantly associated not only with overall risk of HCC (homozygous model, OR = 1.61, 95% CI: 1.40–1.85, P < 0.05; recessive model, OR = 1.40, 95% CI: 1.23–1.59, P < 0.05) but also with the risk of HCC in Chinese patients (homozygous model, OR = 1.78, 95% CI: 1.53–2.08, P < 0.05; recessive model, OR = 1.47, 95% CI: 1.27–1.70, P < 0.05). Limiting the analysis to studies demonstrating Hardy–Weinberg equilibrium (HWE), the results were consistent and robust. Similarly, a significant association between XRCC1 Arg399Gln and HCC risk was found in healthy controls in the general population but not in hospital controls. Trial sequential analysis (TSA), false-positive report probabilities (FPRP), and combined genotype analysis revealed that XRCC1 Arg399Gln is mainly associated with susceptibility to liver cancer. However, there was no association between Arg194Trp or Arg280His and the risk of HCC. These results, indicating that the Arg399Gln polymorphism of XRCC1 is associated with the risk of HCC in the Chinese population, provide a basis for the development of improved detection and treatment approaches.

## Introduction

Hepatocellular carcinoma (HCC) is a primary malignant tumor of the liver that ranks second in cancer deaths in developing countries, sixth in cancer deaths in developed countries [1], and third in the incidence of malignant tumors in China [2]. The onset of HCC is occult, and early symptoms and signs are not easy to detect. Most patients are diagnosed with advanced-stage disease; therefore, treatment is not effective. According to recent epidemiological data, the 5-year survival rate of patients with HCC is only 18% [1]. To improve the prevention and treatment of HCC, it is necessary to clarify its pathogenesis.

The formation of HCC is a multistep process of multiple pathogenies. The causes include chronic hepatitis virus infection, *Aspergillus flavus* toxin damage, long-term drinking, and extensive smoking. However, not everyone exposed to these factors develops HCC. Increasing evidence suggests that HCC is triggered not only by external factors but also by genetic factors.

The base excision repair pathway repairs damaged DNA, thereby maintaining genomic integrity. However, this pathway is prone to errors, resulting in DNA damage and cancer [3]. XRCC1 is a key molecule in the DNA repair process, with a key role in the integrity and stability of the genome and in the pathogenesis and carcinogenesis of various types of tumors. It has been reported that *XRCC1* gene polymorphisms are associated with lung, esophageal, breast, bladder, and gastrointestinal cancer [3–7]. Additionally, a clinical study has shown that *XRCC1* 280 is significantly associated with the number of tumors, tumor size, and tumor location and is an independent risk factor for poor prognosis in patients with HCC [8]. Similarly, *XRCC1* 399 is significantly associated with clinical prognosis. After transcatheter arterial chemoembolization, the risk of death in patients with the A/A+G/A genotype is lower and the median survival time is longer (11.2 months) than those in patients with other genotypes [9].

Many studies have explored the relationship between gene polymorphisms and HCC susceptibility, but a unified conclusion is lacking. In this study, a meta-analysis of studies of XRCC1 Arg194Trp, Arg280His, and Arg399Gln was used to determine the relationship between these polymorphisms and susceptibility to HCC.

## Materials and methods

### Literature search

A comprehensive search was performed against various databases, i.e., PubMed, Web of Science, Cochrane Library, the Chinese National Knowledge Infrastructure, and the Wanfang standard database, to identify case–control studies published through March 1, 2018 that examined the association between *XRCC1* polymorphisms and HCC risk. Searches were performed using various combinations of customized terms and the MeSH-indexed terms “X-ray repair cross complementation group 1” OR “XRCC1” AND “variation” OR “variability” AND “hepatocellular carcinoma” OR “liver cancer”, without restrictions on publication language. The following sequential search strategy was applied for each database: (#1) ‘DNA repair pathway’: ab, ti OR ‘repair gene’/exp ‘OR ‘repair reaction’/exp OR ‘repair response’/exp OR ‘Base Excision Repair/BER’/exp; (#2) ‘X-ray repair cross-complementation group 1’: ab, ti OR ‘XRCC1’: ab, ti OR’ X-ray repair complementing defective repair in Chinese hamster cells 1’/exp; (#3) ‘variation’: ab, ti OR ‘polymorphism’: ab, ti OR ‘SNP’: ab, ti OR ‘Single Nucleotide Polymorphism’/exp OR ‘genetic polymorphism’/exp OR ‘genetic variability’/exp; (#4) ‘liver cancer’: ab, ti OR ‘hepatocellular carcinoma’: ab, ti OR ‘primary hepatic carcinoma’/exp OR ‘primary liver cancer’/exp; (#5) #1 AND #2 AND #3 AND #4.

### Study inclusion and exclusion criteria

#### Inclusion criteria

The inclusion criteria were as follows: (1) studies that examined the association between XRCC1 Arg194Trp, Arg280His, and Arg399Gln and susceptibility to HCC in the Chinese population; (2) studies of humans; (3) case–control studies; (4) studies reporting genotype distributions in the case group and the control group.

#### Exclusion criteria

**Fig 1:**
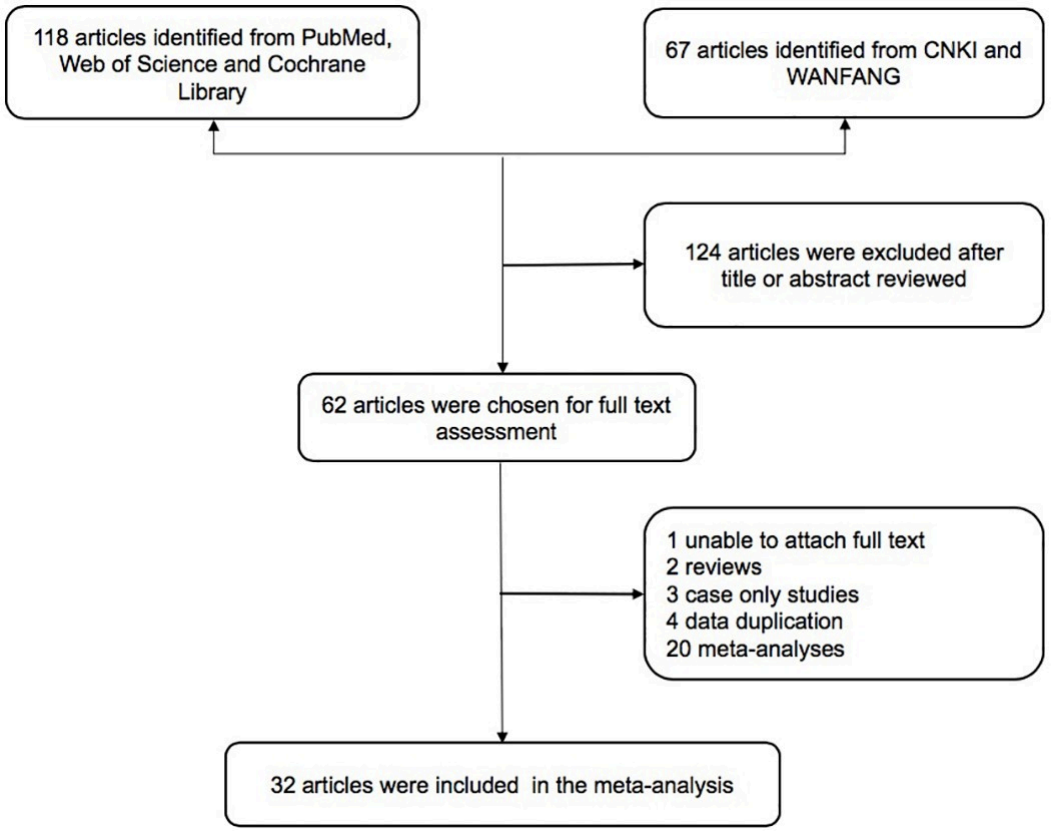
Flow diagram of study selection for the meta-analysis. CNKI, Chinese National Knowledge Infrastructure Database. WFSD, the Wanfang standard database.

The exclusion criteria were as follows: (1) simple case reports, reviews, or commentaries; (2) subjects were single HCC families, animals, or other organisms; (3) the association between XRCC1 Arg194Trp, Arg280His, and Arg399Gln and susceptibility to HCC was not evaluated; (4) data were incomplete; (5) repeated publication (only the most recent or most complete studies were included). The process of literature screening is shown in Fig 1.

The following data were carefully extracted and examined by two assessors: author, publication year, country, case/control number, source, method, Hardy–Weinberg equilibrium (HWE) test, and quality score. The basic features of these studies are shown in Table 1.

**Table 1:** The general data of the observation group and the control group were included in the meta-analysis.

Variable	Years	Country	Cases/Controls	Case			Control			Source	Method	HWE	Score
				c1/c1	c1/c2	c2/c2	c1/c1	c1/c2	c1/c2				
Arg194Trp													
Su [10]	2008	China	100/111	46	50	4	57	43	11	PB	PCR-RFLP	0.50	10
Kiran [11]	2009	India	63/143	8	43	12	27	64	52	PB	PCR-RFLP	0.36	7
Zeng [12]	2010	China	500/507	280	183	37	270	199	38	HB	Taqman	0.87	11
Bo [13]	2011	China	130/130	94	31	5	116	12	2	PB	PCR-RFLP	0.02	9
Tang [14]	2011	China	150/150	94	41	15	81	58	11	PB	PCR-RFLP	0.89	10
Bo [15]	2012a	China	60/60	41	13	6	53	5	2	PB	PCR-RFLP	0.00	8
Han [16]	2012	China	150/158	72	47	31	84	46	28	PB	PCR-RFLP	0.00	8
Yuan [17]	2012a	China	252/250	119	115	18	128	101	21	HB	PCR-RFLP	0.86	9
Zeng [18]	2012	China	46/46	23	23		26	20		HB	PCR-RFLP	—	
Wu [19]	2014	China	218/277	151	55	12	198	68	11	PB	PCR-RFLP	0.10	10
Yang [20]	2015	China	118/120	55	53	10	58	45	17	HB	PCR-RFLP	0.10	5
Krupa [21]	2017	Polish	65/50	57	5	3	41	8	1	HB	Taqman	0.43	5
Guo [22]	2012	China	410/410	264	109	37	292	96	23	HB	PCR-RFLP	0.00	11
Arg280His													
Su [10]	2008	China	100/111	79	20	1	87	21	3	PB	Taqman	0.23	10
Wu [23]	2009	China	100/60	77	22	1	47	13	0	PB	PCR-RFLP	0.34	7
Kiran [11]	2009	India	63/155	19	30	14	91	29	35	PB	PCR-RFLP	0.00	6
Zeng [12]	2010	China	500/507	414	79	7	417	87	3	HB	Taqman	0.50	11
Tang [14]	2011	China	150/150	138	11	1	123	26	1	PB	PCR-RFLP	0.77	10
Han [16]	2012	China	150/158	81	35	34	82	36	40	PB	PCR-RFLP	0.00	8
Yuan [17]	2012a	China	252/250	193	53	6	206	39	5	HB	PCR-RFLP	0.06	9
Yuan [24]	2012b	China	350/400	272	73	5	329	64	7	HB	PCR-RFLP	0.07	10
Bo [15]	2012a	China	60/60	42	12	6	51	6	3	PB	PCR-RFLP	0.00	8
Bo [25]	2012b	China	90/90	64	18	8	78	9	3	PB	PCR-RFLP	0.00	8
Zeng [18]	2012	China	46/46	39	7		35	11		HB	PCR-RFLP	—	
Gulnaz [26]	2013	Pakistan	50/74	24	17	9	44	27	3	HB	PCR-RFLP	0.65	6
He [27]	2015	China	77/40	61	16	0	36	4	0	PB	PCR-RFLP	0.74	7
Krupa [21]	2017	Polish	65/50	57	7	1	36	11	3	HB	Taqman	0.12	5
Arg399Gln													
Yao [28]	2014	China	1486/1996	777	608	101	1437	520	39	PB	PCR-RFLP	0.31	13
Yu [29]	2003	China	577/389	301	223	53	218	143	28	PB	PCR-RFLP	0.50	11
Yang [30]	2004	China	69/136	34	7	28	58	15	63	HB	PCR-RFLP	0.00	7
Long [31]	2004	China	140/536	72	63	5	362	159	15	HB	PCR-RFLP	0.62	10
Kirk [32]	2005	Gambia	149/294	120	26	3	248	43	3	HB	PCR-RFLP	0.46	11
Borentain [33]	2007	France	56/89	27	21	8	27	43	19	PB	Taqman	0.81	8
Ren [34]	2008	China	50/92	32	14	4	46	41	5	PB	PCR-RFLP	0.28	7
Su [10]	2008	China	100/111	40	53	7	69	31	11	PB	Taqman	0.01	9
Kiran [11]	2009	India	63/142	25	33	5	45	70	27	PB	PCR-RFLP	0.98	7
Jia [35]	2010	China	136/136	53	66	17	78	45	13	HB	PCR-RFLP	0.10	10
Zeng [12]	2010	China	500/507	286	180	34	304	167	36	HB	Taqman	0.05	11
Pan [36]	2011	China	202/236	45	105	52	68	112	56	PB	PCR-RFLP	0.46	9
Tang [14]	2011	China	150/150	41	94	15	84	54	12	PB	PCR-RFLP	0.43	10
Guo [22]	2012	China	410/410	203	136	71	227	128	55	PB	PCR-RFLP	0.00	11
He [37]	2012	China	113/113	80	23	10	97	12	4	PB	PCR-RFLP	0.00	10
Han [16]	2012	China	150/158	32	78	40	46	73	39	PB	PCR-RFLP	0.35	9
Bo [15]	2012a	China	60/60	38	14	8	52	5	3	PB	PCR-RFLP	0.00	8
Zeng [18]	2012	China	46/46	33	13		25	21		HB	PCR-RFLP	—	
Mohana [38]	2013	India	93/93	36	45	12	32	51	10	HB	PCR-RFLP	0.12	5
Bose [39]	2013	India	55/209	22	29	4	75	88	46	HB	PCR-RFLP	0.04	8
Gulnaz [24]	2013	Pakistan	50/74	19	14	17	27	32	15	HB	PCR-RFLP	0.34	6
Wu [19]	2014	China	218/277	108	74	36	161	87	29	PB	PCR-RFLP	0.00	9
He [27]	2015	China	77/40	47	26	4	27	12	1	PB	PCR-RFLP	0.80	7
Krupa [21]	2017	Polish	65/50	42	15	8	32	12	6	HB	Taqman	0.02	4
Santonocito [40]	2017	Italia	89/99	37	45	7	59	38	2	HB	PCR	0.14	5
Bazgir [41]	2017	Irania	50/101	12	18	20	31	56	14	HB	PCR-RFLP	0.16	10

Notes: PB, population-based; HB, hospital-based; HWE, Hardy-Weinberg equilibrium; c1:Arg; c2: For Arg194Trp, Trp; ForArg280His, His; ForArg399Gln, Gln.

### Quality assessment

The Newcastle–Ottawa Scale (NOS) was used to evaluate the quality of all eligible studies. The NOS provides a quality rating, ranging from 0 to 10, based on criteria covering three study aspects: study group selection, comparability of cases and controls, and exposure of cases and controls. Results of the quality assessment are shown in Table 2. We also used the quality assessment criteria (S1 Table), derived from a previously published meta-analysis of non-Hodgkin lymphoma [42], for further assessment. Quality scores of studies ranged from 0 to 15. Studies with scores ≤ 9 were considered of low quality, while those with scores > 9 were considered of high quality.

**Table 2:** Results of quality assessment using the Newcastle-Ottawa Scale for cohort studies.

Study (au, year)	A1	A2	A3	A4	B	C1	C2	C3	Score
Su 2008	★	★	★	★	★	★	★	★	8
Kiran 2009	★	★	★	★	★	★	★	一	7
Zeng 2010	★	★	一	★	★	★	★	★	7
Bo 2011	★	★	★	★	★	★	★	一	7
Tang 2011	★	★	★	★	★	★	★	一	7
Bo 2012a	★	★	★	★	★	★	★	一	7
Han 2012	★	★	★	★	★ ★	★	★	★	9
Yuan 2012a	★	★	一	★	★	★	★	★	7
Zeng 2012	★	★	一	★	★	★	★	一	6
Yao 2014	★	★	★	★	★ ★	★	★	一	8
Wu 2014	★	★	★	★	★	★	★	一	7
Yang 2015	★	★	一	★	★	★	★	一	6
Krupa 2017	★	★	一	★	★	★	★	一	6
Wu 2009	★	★	★	★	★	★	★	一	7
Yuan 2012b	★	★	一	★	★	★	★	★	7
Gnlnaz 2013	★	★	一	★	★	★	★	一	6
He 2015	★	★	★	★	★	★	★	★	8
Yu 2003	★	★	★	★	★	★	★	一	7
Yang 2004	★	★	一	★	★ ★	★	★	一	7
Long 2004	★	★	一	★	★	★	★	一	6
Bo 2012b	★	★	★	★	★	★	★	一	7
Kirk 2005	★	★	一	★	★ ★	★	★	一	7
Borentain 2007	★	★	★	★	★	★	★	一	7
Ren 2008	★	★	★	★	★	★	★	一	7
Jia 2010	★	★	一	★	★	★	★	一	6
Pan 2011	★	★	★	★	★ ★	★	★	一	8
Guo 2012	★	★	★	★	★ ★	★	★	一	8
He 2012	★	★	★	★	★	★	★	一	7
Mohana 2013	★	★	一	★	★	★	★	一	6
Bose 2013	★	★	一	★	★ ★	★	★	一	7
Santonocito 2017	★	★	一	★	★ ★	★	★	一	7
Bazgir 2017	★	★	★	★	★	★	★	★	8

Notes: A1, Representativeness of the exposed cohort; A2, Selection of the non-exposed cohort; A3, Ascertainment of exposure; A4, Demonstration that outcome of interest was not present at start of study; B, Comparability of cohorts on the basis of the design or analysis; C1, Assessment of outcome; C2, Was follow-up long enough for outcomes to occur; C3, Adequacy of follow up of cohorts; A, B, C represent Selection, Comparability, Outcome, respectively;★and★★indicate compliance with the requirements of the definition, for which specific meaning see S1 Text.

### Data analysis

RevMan 5.3 was used for meta-analysis, and Q and I^2^ values were used to evaluate heterogeneity. If the heterogeneity test showed P > 0.1 or I^2^ < 50%, a fixed effect model was used, and heterogeneity was considered relatively low; if P < 0.1 or I^2^ > 50%, heterogeneity was considered high, and a subgroup analysis or sensitivity analysis was used to study the source of heterogeneity. The odds ratio (OR) and 95% confidence interval (CI) were used as indicators of the effect for each result. HWE was calculated using HWSIM (http://krunch.med.yale.edu/hwsim/website). P > 0.05 indicated equilibrium; P < 0.05 indicated a departure from HWE. A sensitivity analysis was used to evaluate the stability of the results. False-positive report probabilities (FPRP) were calculated using the FPRP calculation spreadsheet (see http://jncicancerspectrum.oupjournals.org/jnci-/content/vol96/issue6) to assess positive results. The FPRP threshold was set to 0.2, and the prior probability was set to 0.1 to detect the OR. A significant result with an FPRP value of less than 0.2 indicated a notable finding. All statistical tests were two-sided, and P < 0.05 was considered statistically significant. Trial sequential analysis (TSA) was used to reduce random errors and increase the robustness of the conclusions, using a 5% significance level for type I errors and a 20% significance level for type II errors, and the amount of information and a TSA monitoring boundary were determined.

## Results

### Study selection and characteristics

The meta-analysis was conducted according to the recommendations of the “Preferred Reporting Items for Systematic Reviews and Meta-Analyses” (PRISMA) statement (S1 Checklist) and “Meta-analysis on Genetic Association Studies” statement (S2 Checklist). Systemic literature searches identified 32 articles [10–41], eight of which discussed all three loci, four of which discussed two loci, and twenty of which discussed a single locus. Of the 32 studies, 13 analyzed the XRCC1 Arg194Trp polymorphism, 14 analyzed Arg280His, and 26 analyzed Arg399Gln.

The XRCC1 Arg194Trp, Arg280His, and Arg399Gln polymorphisms were evaluated by calculating ORs and 95% CIs under homozygous, heterozygous, dominant, and recessive models. The results are summarized in Table 3.

**Table 3:** Overall and subgroup analysis of the XRCC1 polymorphisms and cancer risk.

Varible	N	Homozygous genetic model			Heterozygous genetic model			Dominant genetic model			Recessive genetic model		
		OR(95%CI)	Phet	I^2^	OR(95%CI)	Phet	I^2^	OR(95%CI)	Phet	I^2^	OR(95%CI)	Phet	I^2^
Arg194Trp		Trp/Trp vs Arg/Arg			Trp/Trp vs Arg/Trp			Trp/Trp + Arg/Trp vs Arg/Arg			Trp/Trp vs Arg/Arg + Arg/Trp		
All	13	1.13(0.90,1.41)	0.34	10	1.42(1.24,1.62)	0.01	57	1.14(1.01,1.29)	0.01	53	1.02(0.82,1.26)	0.05	44
All-China	11	1.14(0.91,1.44)	0.25	21	1.41(1.23,1.61)	0.03	52	1.15(1.01,1.30)	0.01	58	1.11(0.89,1.39)	0.20	27
All-HWE	8	0.92(0.69,1.21)	0.78	0	1.39(1.18,1.64)	0.01	60	1.00(0.87,1.16)	0.52	0	0.82(0.63,1.06)	0.15	35
All-HWE-China	6	0.91(0.68,1.23)	0.64	0	1.38(1.16,1.62)	0.06	53	0.99(0.85,1.15)	0.44	0	0.91(0.68,1.21)	0.33	13
All-PB	7	1.18(0.84,1.67)	0.37	8	1.72(1.37,2.14)	0.01	68	1.26(1.03,1.54)	0.01	68	0.97(0.71,1.33)	0.04	55
All-HB	6	1.09(0.82,1.47)	0.23	29	1.26(1.07,1.50)	0.50	0	1.08(0.94,1.26)	0.37	14	1.06(0.79,1.40)	0.20	34
Arg280His		His/His vs Arg/Arg			His/His vs Arg/His			His/His + Arg/His vs Arg/Arg			His/His vs Arg/Arg + Arg/His		
All	13	1.43(0.91,2.25)	0.15	31	1.20(1.02,1.41)	0.00	69	1.19(1.02,1.38)	0.00	67	1.15(0.84,1.56)	0.22	23
All-China	10	1.14(0.77,1.69)	0.47	0	1.14(0.96,1.35)	0.03	53	1.13(0.96,1.33)	0.01	56	1.10(0.76,1.59)	0.51	0
All-HWE	9	1.28(0.61,2.67)	0.18	33	1.05(0.88,1.26)	0.03	53	1.08(0.91,1.28)	0.02	57	1.31(0.77,2.23)	0.17	34
All-HWE-China	7	1.15(0.59,2.22)	0.68	0	1.08(0.90,1.31)	0.03	56	1.10(0.91,1.32)	0.05	52	1.09(0.58,2.05)	0.59	0
All-PB	7	1.37(0.80,2.35)	0.24	26	1.34(1.03,1.73)	0.00	77	1.30(1.03,1.64)	0.00	75	1.03(0.71,1.48)	0.45	0
All-HB	6	1.47(0.62,3.47)	0.11	46	1.12(0.91,1.37)	0.11	47	1.12(0.92,1.36)	0.06	53	1.49(0.84,2.63)	0.12	45
Arg399Gln		Gln/Gln vs Arg/Arg			Gln/Gln vs Arg/Gln			Gln/Gln + Arg/Gln vs Arg/Arg			Gln/Gln vs Arg/Arg + Arg/Gln		
All	25	1.61(1.40,1.85)	0.00	69	1.55(1.42,1.68)	0.00	74	1.56(1.45,1.69)	0.00	79	1.40(1.23,1.59)	0.00	64
All-China	17	1.78(1.53,2.08)	0.00	67	1.66(1.52,1.82)	0.00	77	1.68(1.54,1.82)	0.00	81	1.47(1.27,1.70)	0.00	60
All-HWE	17	1.80(1.51,2.13)	0.00	72	1.58(1.44,1.73)	0.00	79	1.64(1.50,1.79)	0.00	82	1.53(1.30,1.79)	0.00	67
All-HWE-China	10	2.00(1.65,2.42)	0.00	73	1.71(1.55,1.89)	0.00	82	1.77(1.61,1.95)	0.00	84	1.57(1.31,1.87)	0.00	69
All-PB	14	1.83(1.55,2.17)	0.00	74	1.66(1.51,1.83)	0.00	81	1.73(1.57,1.90)	0.00	84	1.51(1.29,1.77)	0.00	69
All-HB	11	1.19(0.93,1.53)	0.00	74	1.29(1.10,1.50)	0.04	47	1.24(1.07,1.43)	0.00	57	1.17(0.92,1.48)	0.01	58

### Quantitative synthesis

**Fig 2:**
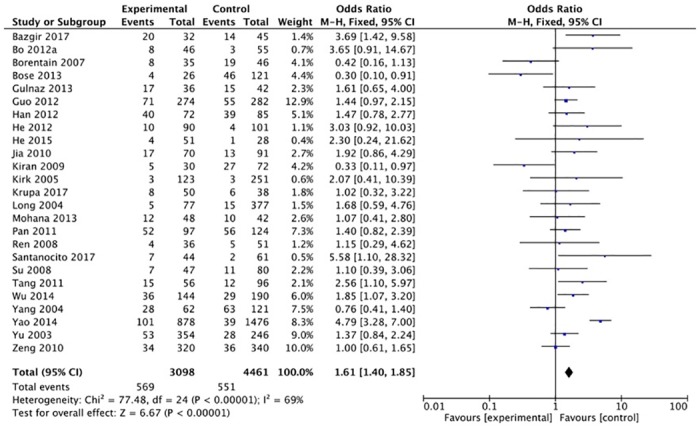
Forest plot of the association between XRCC1 Arg399Gln polymorphism and HCC risk under a homozygous model.

**Fig 3:**
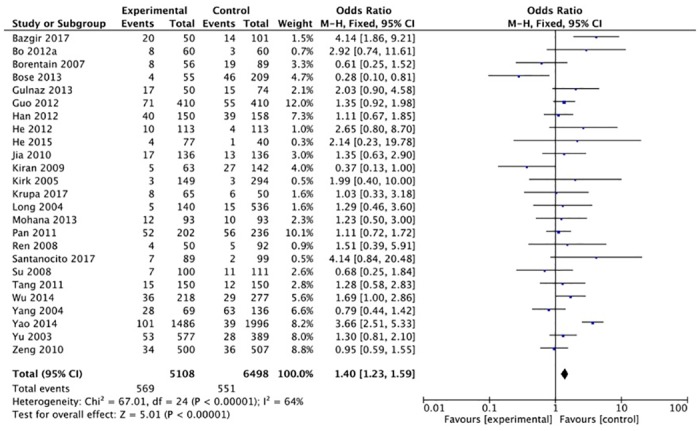
Forest plot of the association between XRCC1 Arg399Gln polymorphism and HCC risk under a recessive model.

**Fig 4:**
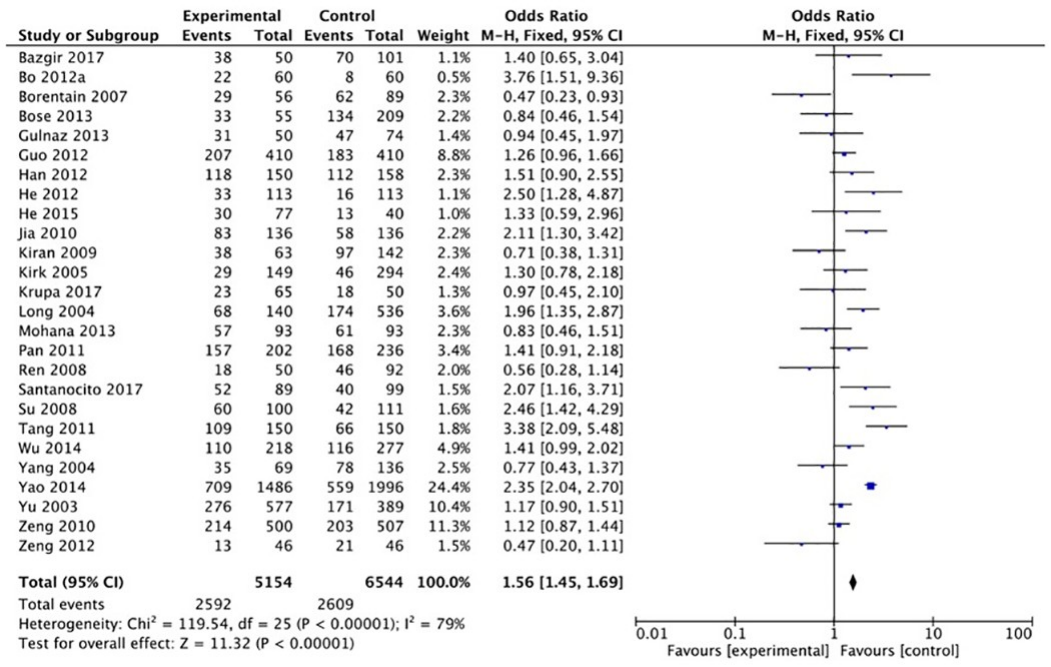
Forest plot of the association between XRCC1 Arg399Gln polymorphism and HCC risk under a dominant model.

**Fig 5:**
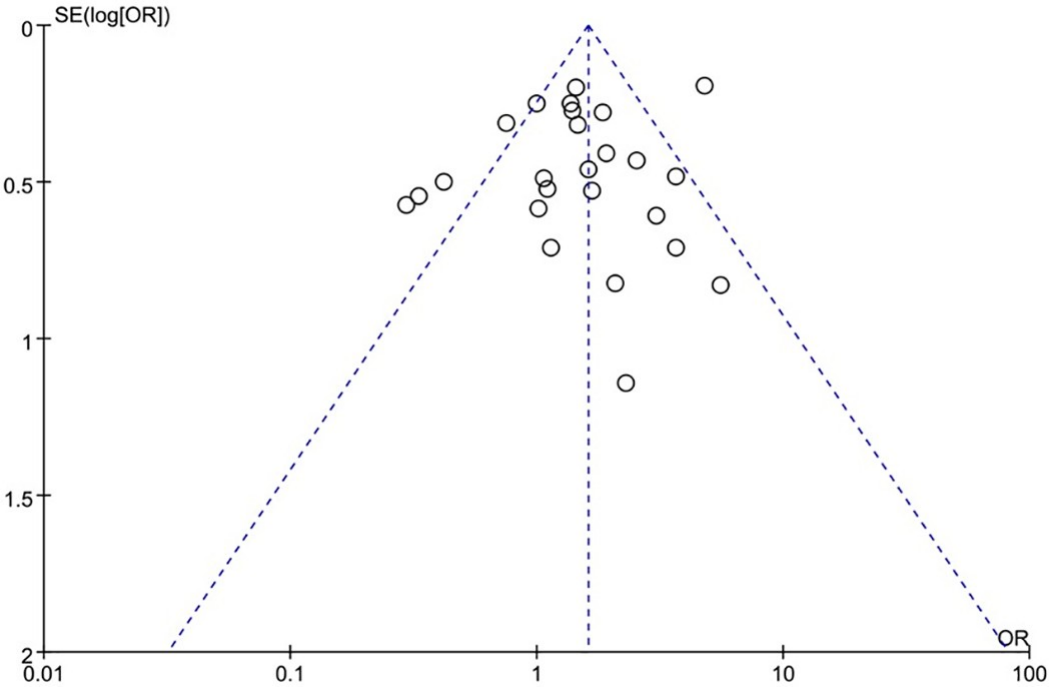
Funnel plot to detect publication bias in data on XRCC1 Arg399Gln polymorphism according to a homozygous model.

**Fig 6:**
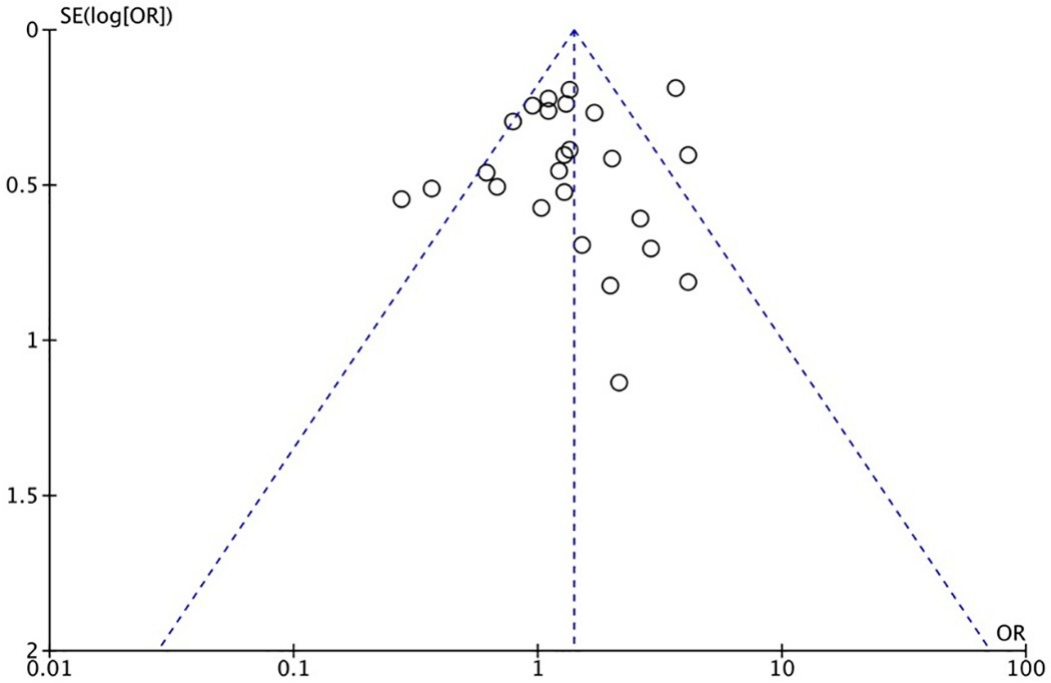
Funnel plot to detect publication bias in data on XRCC1 Arg399Gln polymorphism according to a recessive model.

**Fig 7:**
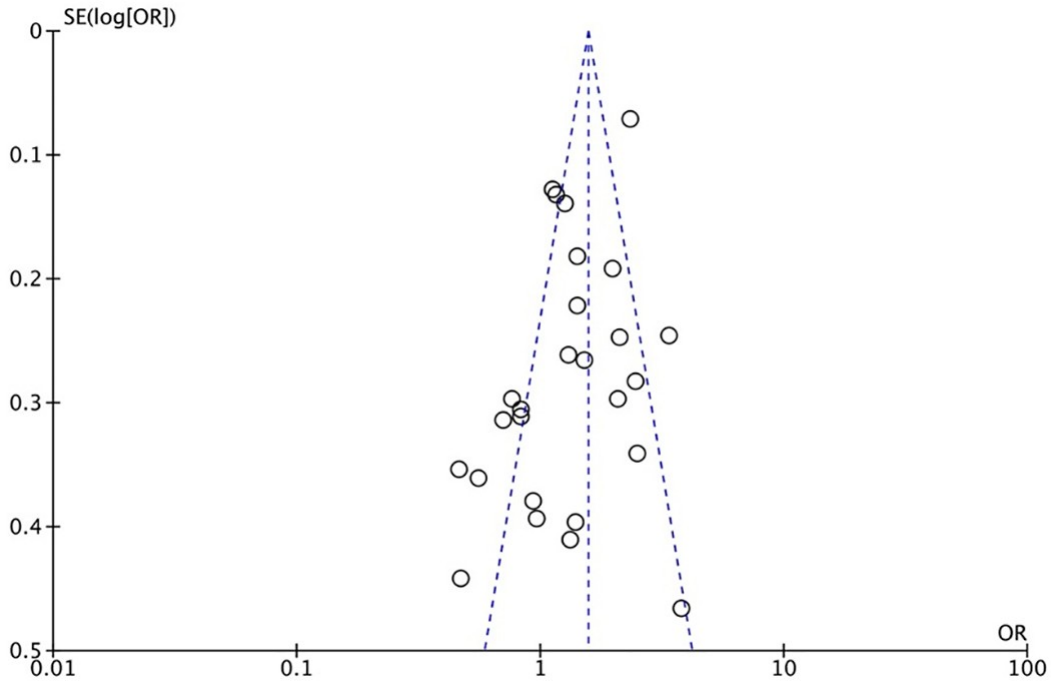
Funnel plot to detect publication bias in data on XRCC1 Arg399Gln polymorphism according to a dominant model.

The XRCC1 Arg399Gln polymorphism was related to the risk of HCC in the Arg399Gln homozygous genetic model (OR = 1.61, 95% CI: 1.40–1.85, P_heterogeneity_ < 0.05; Fig 2), recessive genetic model (OR = 1.40, 95% CI: 1.23–1.59, P_heterogeneity_ < 0.05; Fig 3), dominant genetic model (OR = 1.56, 95% CI: 1.45–1.69, P_heterogeneity_ < 0.05; Fig 4), and heterozygous genetic model (OR = 1.55, 95% CI: 1.42–1.68, P_heterogeneity_ < 0.05). Arg399Gln was also associated with susceptibility to HCC in the Chinese population based on the homozygous genetic model (OR = 1.78, 95% CI: 1.53–2.08, P_heterogeneity_ < 0.05) and recessive genetic model (OR = 1.47, 95% CI: 1.27–1.70, P_heterogeneity_ < 0.05), suggesting that Gln/Gln is a risk factor for HCC. Limiting the analysis to studies demonstrating HWE, inconsistent results were obtained (Table 3). In the Indian population, the Arg399Gln homozygous genetic model (OR = 0.49, 95% CI: 0.27–0.87, P_heterogeneity_ = 0.15) and recessive genetic model (OR = 0.51, 95% CI: 0.30–0.87, P_heterogeneity_ = 0.07) indicated that Gln/Gln is a protective factor for liver cancer. Similarly, the funnel plot for Arg399Gln was asymmetric, implying a slight publication bias (Fig 5, funnel plot for the Arg399Gln homozygous model; Fig 6, funnel plot for the Arg399Gln recessive model; Fig 7, funnel plot for the Arg399Gln dominant model).

The XRCC1 Arg280His polymorphism was not associated with the risk of HCC under the Arg280His homozygous genetic model (OR = 1.43, 95% CI: 0.91–2.25, P_heterogeneity_ = 0.15), recessive genetic model (OR = 1.15, 95% CI: 0.84–1.56, P_heterogeneity_ = 0.22), dominant genetic model (OR = 1.19, 95% CI: 1.02–1.38, P_heterogeneity_ < 0.01), or heterozygous genetic model (OR = 1.20, 95% CI: 1.02–1.41, P_heterogeneity_ < 0.01). In addition, no association was observed for any subgroups.

Similarly, the XRCC1 Arg194Trp was not related to susceptibility to HCC under the Arg194Trp homozygous genetic model (OR = 1.13, 95% CI: 0.90–1.41, P_heterogeneity_ = 0.34), recessive genetic model (OR = 1.02, 95% CI: 0.82–1.26, P_heterogeneity_ = 0.05), dominant genetic model (OR = 1.14, 95% CI: 1.01–1.29, P_heterogeneity_ < 0.05), or heterozygous genetic model (OR = 1.42, 95% CI: 1.24–1.62, P_heterogeneity_ < 0.05). No significant association was found in any of the subgroups.

### Sensitivity analysis

Owing to the slight heterogeneity of the results for Arg194Trp, a sensitivity analysis was performed, which indicated that Tang et al. [14] was the source of the heterogeneity. After eliminating this study, Arg194Trp was unrelated to susceptibility to HCC based on the homozygous genetic model (OR = 1.13, 95% CI: 0.78–1.64, P_heterogeneity_ = 0.55), heterozygous genetic model (OR = 1.17, 95% CI: 0.97–1.39, P_heterogeneity_ < 0.01), dominant genetic model (OR = 1.14, 95% CI: 0.96–1.35, P_heterogeneity_ < 0.01), and recessive genetic model (OR = 0.96, 95% CI: 0.67–1.36, P = 0.79).

The full analysis suggested that Arg280His was not associated with susceptibility to HCC under the homozygous genetic model (OR = 1.56, 95% CI: 1.11–2.18, P_heterogeneity_ = 0.19) or recessive genetic model (OR = 1.45, 95% CI: 1.05–1.99, P_heterogeneity_ = 0.07). However, many studies had small sample sizes. After those with N < 200 were eliminated, the Arg280His homozygous genetic model (OR = 1.13, 95% CI: 0.78–1.64, P_heterogeneity_ = 0.55), heterozygous genetic model (OR = 1.17, 95% CI: 0.97–1.39, P_heterogeneity_ < 0.01), dominant genetic model (OR = 1.14, 95% CI: 0.96–1.35, P_heterogeneity_ < 0.01), and recessive genetic model (OR = 0.96, 95% CI: 0.67–1.36, P_heterogeneity_ = 0.79), still indicated a lack of evidence for an association with susceptibility to HCC.

### TSA, combined genotype analysis, and FPRP analysis

**Fig 8:**
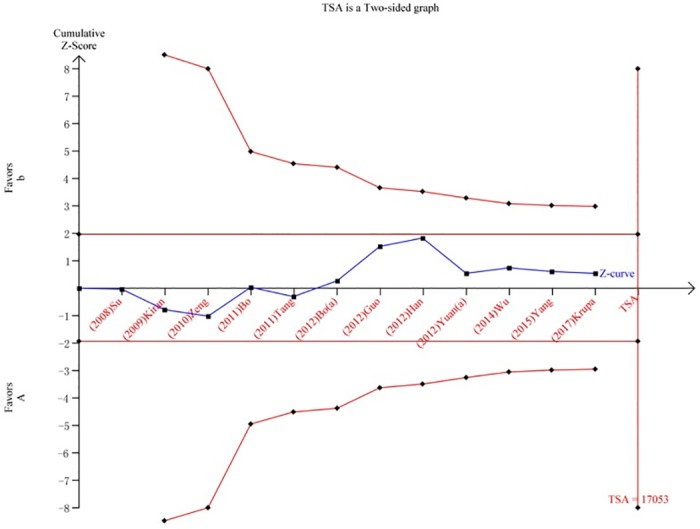
Trial sequential analysis for XRCC1 Arg194Trp gene polymorphism under the allele contrast model.

**Fig 9:**
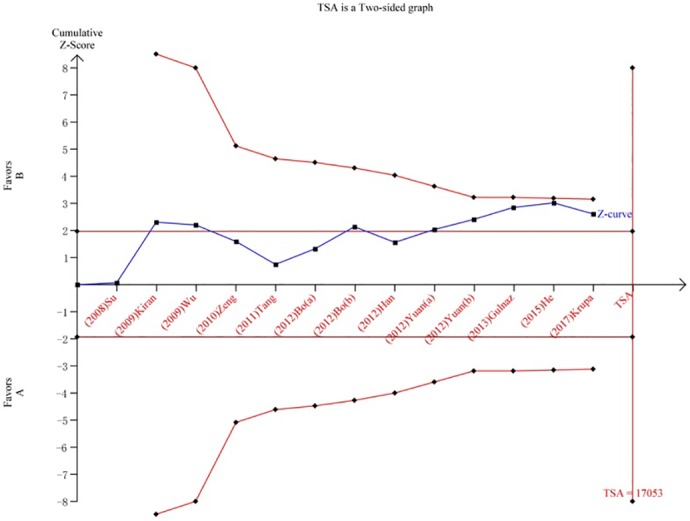
Trial sequential analysis for XRCC1 Arg280His gene polymorphism under the allele contrast model.

**Fig 10:**
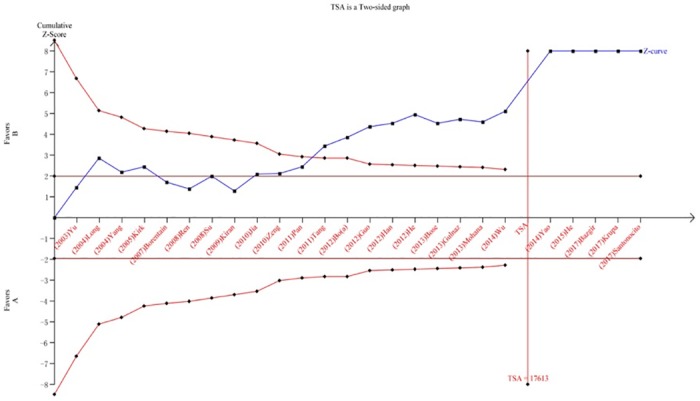
Trial sequential analysis for XRCC1 Arg399Gln gene polymorphism under the allele contrast model.

We performed a TSA to reduce random errors and increase the robustness of the conclusions. The TSA of the Arg194Trp polymorphism model showed that the cumulative z-curve did not cross the traditional cut-off value, nor did it cross the TSA threshold. Moreover, the expected amount of information was not obtained, indicating that the difference in the XRCC1 Arg194Trp polymorphism between the HCC group and the control group was not statistically significant and that additional experiments are needed (Fig 8). The TSA of the allele models for the Arg280His polymorphism showed that the cumulative z-curve crossed the traditional cut-off value but did not cross the TSA threshold, and the cumulative amount of information was insufficient (Fig 9). The TSA of the allele models for the Arg399Gln polymorphism showed that the cumulative z-curve crossed both the traditional threshold and the TSA threshold, and the accumulated information was sufficient, indicating that no further evidence was needed to verify the conclusion (Fig 10).

HWE-based studies were performed using the combined genotype analysis. XRCC1 Arg194Trp + Arg280His was not associated with HCC susceptibility under the homozygous genetic model (OR = 1.00, 95% CI: 0.78–1.29) or recessive genetic model (OR = 0.90, 95% CI: 0.71–1.14). When Arg399Gln was combined with either or both of the other polymorphisms, correlations with HCC susceptibility were detected, indicating that the main SNP related to HCC risk is XRCC1 Arg399Gln (Table 4).

**Table 4:** Combined genotype analysis for three XRCC1 single nucleotide polymorphisms.

All-HWE	Homozygous genetic model	Heterozygous genetic model	Dominant genetic model	Recessive genetic model
	OR(95%CI)	OR(95%CI)	OR(95%CI)	OR(95%CI)
Arg194Trp + Arg280His	0.96(0.73,1.26)	1.22(1.08,1.38)	1.03(0.93,1.15)	0.90(0.71, 1.14)
Arg194Trp + Arg399Gln	1.50(1.29,1.74)	1.53(1.41,1.66)	1.42(1.32,1.53)	1.29(1.12,1.48)
Arg280His + Arg399Gln	1.77(1.49,2.10)	1.45(1.33,1.57)	1.50(1.39,1.62)	1.51(1.29,1.76)
Arg194Trp + Arg280His + Arg399Gln	1.49(1.29,1.73)	1.43(1.33,1.54)	1.36(1.27,1.46)	1.29(1.13,1.47)

Table 5 shows the FPRP values for our positive results using different prior probability levels. Assuming a prior probability of 0.1 and a specific genotype with an OR of 1.5, the statistical power was 0.856, and the FPRP value was < 0.001 for the recessive model of the XRCC1 Arg399Gln polymorphism. Thus, the risk of liver cancer was elevated for all individuals. In addition, the FPRP values for the Chinese population, the all-HWE-compliant population, the China-HWE-compliant subgroup, and the population-based (PB) group were all less than 0.2, indicating reliable results.

**Table 5:** False-positive report probability values for associations between the risk of hepatocellular carcinoma and the frequency of genotypes of XRCC1 gene.

Arg399Gln Homozygous	Crude OR(95%CI)	Statistical power	P-value	Prior probability				
				0.25	0.1	0.01	0.001	0.0001
All	1.61(1.40,1.85)	0.159	0.000	**0.000**	**0.000**	**0.000**	**0.000**	**0.000**
All-China	1.78(1.53,2.08)	0.016	0.000	**0.000**	**0.000**	**0.000**	**0.000**	**0.000**
All-HWE	1.80(1.51,2.13)	0.174	0.000	**0.041**	**0.114**	0.585	0.934	0.993
All-HWE-China	2.00(1.65,2.42)	0.002	0.000	**0.000**	**0.000**	**0.000**	**0.000**	**0.000**
All-PB	1.83(1.55,2.17)	0.011	0.000	**0.000**	**0.000**	**0.000**	**0.000**	**0.000**
Arg399Gln Recessive								
All	1.40(1.23,1.59)	0.856	0.000	**0.000**	**0.000**	**0.004**	**0.003**	0.025
All-China	1.47(1.27,1.70)	0.607	0.000	**0.000**	**0.000**	**0.000**	**0.000**	**0.003**
All-HWE	1.53(1.30,1.79)	0.402	0.000	**0.000**	**0.000**	**0.000**	**0.000**	**0.003**
All-HWE-China	1.57(1.31,1.87)	0.305	0.000	**0.000**	**0.000**	**0.000**	**0.001**	**0.014**
All-PB	1.51(1.29,1.77)	0.467	0.000	**0.000**	**0.000**	**0.000**	**0.001**	**0.008**

## Discussion

HCC is a serious digestive system tumor that is typically detected at an advanced stage, when treatment approaches are limited and prognosis is poor. Studies have shown that bad eating habits, alcohol consumption, environment, work stress, and emotional changes are risk factors for HCC in high-incidence areas. However, not all individuals exposed to these risk factors develop HCC, indicating that genetic susceptibility may be important. Defects or inadequate DNA repair caused by polymorphisms in DNA repair genes increase the risk of cancer. Previous studies have reported that *XRCC1* expression is elevated in colorectal [43], esophageal [44], and lung cancer tissues [45]. Similarly, Krupa et al. [21] showed that the mRNA expression of *XRCC1* in HCC tissues was significantly lower than that in adjacent non-lesional tissues. The expression of *XRCC1* in cancer tissues is closely related to the intrinsic genetic phenotype. Thus, DNA repair gene polymorphisms may play an important role in susceptibility to liver cancer.

Many studies have shown that the XRCC1 Arg399Gln polymorphism is associated with HCC risk, while susceptibility is lower for carriers of Arg194Trp and Arg280His; however, the results of previous studies have been inconsistent. Guo et al. [22] found that, compared with Arg/Arg, XRCC1 194Trp/Trp was significantly associated with risk of HCC, and individuals carrying XRCC1 399Gln/Gln showed an increased risk of HCC. However, Yang et al. [30], found that this polymorphism was not related to HCC risk. Su et al. [10] suggested that the Arg194Trp and Arg280His polymorphisms are not related to susceptibility to HCC but that the Arg399Gln polymorphism is a susceptibility factor for HCC, with Gln/Gln as a risk factor, consistent with the results of this study. Jia et al. [35] found that the XRCC1 399 Arg/Gln genotype conferred increased HCC risk. Han et al. [16] found that the median survival rate of individuals carrying the XRCC1 Gln/Gln genotype was significantly lower than that of individuals carrying the XRCC1 Arg/Arg genotype. However, Zeng et al. [12] suggested that XRCC1 Arg194Trp, Arg280His, and Arg399Gln are not predisposing factors for HCC but found that there was an additive interaction between *XRCC1* polymorphisms and smoking, drinking, and chronic hepatitis B virus (HBV) infection. Similarly, Yuan et al. [17] found that XRCC1 Arg194Trp and Arg280His are not associated with the risk of HCC but that Arg399Gln is associated with a significantly increased risk of HCC in the background of HBV infection and family history.

In a previous meta-analysis, Xu et al. [46] found that Arg280His was associated with the risk of HCC and that His/His increases the risk of disease. Li et al. [47] found that Arg194Trp and Arg280His were not related to the risk of HCC, but that 399 Arg/Gln was significantly associated with the risk of HCC, and the results were still significant in studies demonstrating HWE. Similarly, Liu et al. [48] indicated that there was a significant correlation between Arg399Gln and susceptibility to HCC in the Chinese population. Shi et al. [49] found that 399 Arg/Gln was unrelated to HCC but that it was significantly correlated with the incidence of HCC in southern China, suggesting that there was genetic heterogeneity. In our comprehensive meta-analysis of Arg194Trp, Arg280His, and Arg399Gln case–control studies, we grouped the populations that satisfied HWE, calculated FPRPs, and performed a TSA to increase the robustness of the conclusions. Our findings showed that the Arg399Gln polymorphism increased susceptibility to liver cancer, while Arg194Trp and Arg280His were not associated with susceptibility to liver cancer. However, additional samples are needed to further evaluate these findings.

Zhu et al. [50] studied the relationship between the *XPC* genotype and DNA repair ability in an alkaline comet assay challenged by benzo[a]pyrene diol epoxide (BPDE) and γ radiation. Healthy subjects with the XPC Lys939Gln variant allele (AC and CC) were found to have significantly increased rates of DNA damage induced by BPDE and γ irradiation compared to homozygous wild-type (AA) subjects. In contrast, subjects with the Ala499Val variant allele (CT and TT) showed reduced BPDE- and γ radiation-induced DNA damage. Reinardy et al. [51] evaluated echinoderms after 24 h of exposure to genotoxic agents (UV-C, hydrogen peroxide, and bleomycin) and found that adult sea urchin coelomocytes and larvae with *XRCC1* polymorphisms showed more mutations in the body cavity after recovery, indicating the heterogeneous response of echinoderms to DNA damage and revealing that DNA repair ability within host cells may be regulated by specific gene polymorphisms. Therefore, XRCC1 plays a crucial role in maintaining genomic stability and preventing cancer. We hypothesize that people exposed to risk factors for liver cancer are more likely to develop *XRCC1* mutations, resulting in an altered DNA repair capacity and increased susceptibility to liver cancer. In addition, Kuptsova et al. [52] found that after a standard chemotherapy induction regimen in elderly patients with acute myeloid leukemia, different DNA repair gene variants repaired chemotherapy-induced DNA damage, which may affect drug toxicity and treatment response to varying degrees. Xuan et al. [53] found that XRCC1 can increase the base repair ability and promote tumor resistance via the tumor drug resistance pathway, suggesting that variants in the DNA repair pathway may impact patient outcomes and treatment-related responses. Wang et al. [54] found that XRCC1 protein levels are significantly down-regulated in gastric cancer lesions compared with levels in adjacent non-cancerous tissues in a study of the prognosis and predictive role of XRCC1 in patients treated with surgery alone or in combination with adjuvant chemotherapy. Low expression of XRCC1 was significantly associated with shorter overall survival and clinicopathological features of unassisted patients. The prognosis of patients treated with adjuvant fluorouracil-leucovorin-oxaliplatin was significantly improved compared with that for surgery alone. However, this effect was only significant in the low expression group; therefore, XRCC1 protein expression in tumors is a novel candidate prognostic marker and response predictor. Li et al. [55] performed a prognostic analysis of 150 patients with HCC and found that patients carrying the Gln/Gln genotype showed a significantly lower median survival than individuals with the Arg/Arg genotype. Compared with Arg/Arg carriers, the median survival rate of Arg/Gln + Gln/Gln carriers was reduced. Therefore, we hypothesize that the XRCC1 Gln/Gln genotype can be used as a negative indicator in liver cancer and that XRCC1 can serve as a potential indicator for clinical diagnosis and prognosis, as well as a new potential target for clinical treatment in HCC cases.

Our study had some limitations. We observed high heterogeneity among studies, which may be related to the choice of the control population, differences in living environments, and differences in family genetic background. Second, many studies included in the analysis had small sample sizes. To ensure the stability of the results, we evaluated the FPRP and performed a TSA. In addition, we detected a slight publication bias, suggesting that additional well-designed studies are needed. Our results showed that the XRCC1 Arg399Gln Gln/Gln genotype is a risk factor for liver cancer in the Chinese population.

## Conclusions

*XRCC1* polymorphisms are still a major topic in cancer research. Previous meta-analyses of these polymorphisms have yielded inconsistent results. In this study, relevant literature was obtained to resolve this controversy. Our results indicated that XRCC1 Arg399Gln is significantly associated with the risk of HCC, especially in the Chinese population. In addition, there was a slight publication bias, suggesting the need for further research.

## Supporting information

S1 Checklist PRISMA 2009 checklist. (DOC)

S2 Checklist Meta-analysis of Genetic Association Studies checklist. (DOCX)

S1 Text. Nosgen The Newcastle-Ottawa Scale (NOS) as a quality assessment method in this systematic review. (PDF)

S1 Table Score of quality assessment. (DOC)

S2 Table Gene polymorphisms distribution of XRCC1 gene polymorphic sites in HCC group and control group. (DOC)

S1 Appendix The detailed reasons for excluded articles. (DOC)

S2 Appendix The differences from the previous meta-analyses. (DOCX)

S3 Appendix The detailed search criteria and the full data range for the search in each database. (DOC)
