# Supplementary material for: Associations between three XRCC1 polymorphisms and hepatocellular carcinoma risk: A meta-analysis of case-control studies
Source: PLoS One. 2018 Nov 8;13(11):e0206853. doi: 10.1371/journal.pone.0206853 (PMC6226104; doi:10.1371/journal.pone.0206853)
Supplement: S2 Table — (DOC) [file pone.0206853.s005.doc]

Gene polymorphisms distribution of XRCC1 gene polymorphic sites in HCC group and control group n( % ).

1. Su HY, Xu K. A case-control study on association between genetic polymorphisms of DNA repair and hepatic cell cancer susceptibility. China Medical University. 2008

| Arg194Trp | Arg/Arg (%) | Arg/Trp (%) | Trp/Trp (%) | P-values |
| --- | --- | --- | --- | --- |
| Cases | 46 (46.00) | 50 (50.00) | 4 (4.00) |  |
| Controls | 57 (51.35) | 43 (38.74) | 11 (9.91) | 0.110 |
| Arg280His | Arg/Arg (%) | Arg/His (%) | His/His (%) | P-values |
| Cases | 79 (79.00) | 20 (20.00) | 1 (0.00) |  |
| Controls | 87 (78.38) | 21 (18.92) | 3 (2.70) | 0.912 |
| Arg399Gln | Arg/Arg (%) | Arg/Gln (%) | Gln/Gln (%) | P-values |
| Cases | 40 (40.00) | 53 (53.00) | 7 (7.00) |  |
| Controls | 69 (62.16) | 31 (27.93) | 11 (9.91) | 0.001 |

2. Kiran M, Saxena R, Chawla YK, Kaur J. Polymorphism of DNA repair gene XRCC1 and hepatitis-related hepatocellular carcinoma risk in Indian population. [Mol Cell Biochem.](http://nc.yuntsg.com/show.do?q=Polymorphism%20of%20DNA%20repair%20gene%20XRCC1%20and%20hepatitis-related%20hepatocellular%20carcinoma%20risk%20in%20Indian%20population%5bTitle%5d) 2009; 327:7-13. Epub 2009/02/05. PMID: 19194663. DOI: [10.1007/s11010-009-0035-3](http://doi.org/10.1007/s11010-009-0035-3).

| Arg194Trp | Arg/Arg (%) | Arg/Trp (%) | Trp/Trp (%) |
| --- | --- | --- | --- |
| Cases | 8 (12.69) | 43 (68.25) | 12 (19.05) |
| Controls | 27 (18.88) | 64 (44.75) | 52 (36.36) |
| Arg280His | Arg/Arg (%) | Arg/His (%) | His /His (%) |
| Cases | 19 (30.16) | 30 (47.62) | 14 (22.22) |
| Controls | 91 (58.70) | 29 (18.71) | 35 (22.58) |
| Arg399Gln | Arg/Arg (%) | Arg/Gln (%) | Gln/Gln (%) |
| Cases | 25 (39.68) | 33 (52.38) | 5 (7.94) |
| Controls | 45 (31.69) | 70 (49.29) | 27 (19.01) |

3. Zeng XY, Yu HP, Qiu XQ, Ji L, Li LM. A case-control study of polymorphism of XRCC1 gene and the risk of hepatocellular carcinoma. Chin J Dis Control Prev. 2010;14(8): 760-763.

Case: n=500; Control: n=507.

| Arg194Trp | Arg/Arg (%) | Arg/Trp (%) | Trp/Trp (%) |
| --- | --- | --- | --- |
| Cases | 56.0 % | 36.6 % | 7.4 % |
| Controls | 53.3 % | 39.3 % | 7.4 % |
| Arg280His | Arg/Arg (%) | Arg/His (%) | His/His (%) |
| Cases | 82.8 % | 15.8 % | 1.4 % |
| Controls | 82.2 % | 17.2 % | 0.6 % |
| Arg399Gln | Arg/Arg (%) | Arg/Gln (%) | Gln/Gln (%) |
| Cases | 57.2 % | 36.0% | 6.8 % |
| Controls | 60.0 % | 32.9 % | 7.1 % |

4. Bo WL, Zhang GX, Li DY, Wang X, Liang T. Polymorphisms of DNA repair gene XRCC1 and susceptibility to hepatic cancer. Xiandai Zhongliu Yixue. 2011;19(09): 1724-1726.

| Arg194Trp | Liver cancer group | Control group | P |
| --- | --- | --- | --- |
| Arg/Arg (%) | 94 (72. 31) | 116 (89. 23) | p>0.05 |
| Arg/Trp (%) | 31 (23.85) | 12 (9.23) | P<0.05 |
| Trp/Trp (%) | 5 (3.84) | 2 (1.54) | P<0.05 |

5. Tang YT, Li XP, Liu TQ, Yang JR, Luo JQ, Liang ZX. Genetic polymorphisms of DNA repair genes in patients with hepatocellular carcinoma. Shandong Yiyao. 2011;51(42): 19-20.

| Arg194Trp | Arg/Arg | Arg/Trp | Trp/Trp |
| --- | --- | --- | --- |
| Case | 94 | 41 | 15 |
| Control | 81 | 58 | 11 |
| Arg280His | Arg/Arg | Arg/His | His /His |
| Case | 138 | 11 | 1 |
| Control | 123 | 26 | 1 |
| Arg399Gln | Arg/Arg | Arg/Gln | Gln/Gln |
| Case | 41 | 94 | 15 |
| Control | 84 | 54 | 12 |

6. Bo WL, Zhang GX, Li DY, Wang X. The study of Polymorphisms of DNA Repair Gene XRCC1 and Hepatic Cancer. Zhongguo Xian Dai Yi Xue Za Zhi. 2012;22(18): 45-48.

| Genotype | Liver cancer group (n=60) | Control group  (n=60) | P |
| --- | --- | --- | --- |
| Arg194Trp |  |  |  |
| Arg/Arg (%) | 41 (68.33) | 53 (88.33) | p>0.05 |
| Arg/Trp (%) | 13 (21.67) | 5 (8.33) | P<0.05 |
| Trp/Trp (%) | 6 (10.00) | 2 (3.33) | P<0.05 |
| Arg280His |  |  |  |
| Arg/Arg (%) | 42 (70.00) | 51 (85.0) | p>0.05 |
| Arg/His (%) | 12 (20.00) | 6 (10.00) | P<0.05 |
| His /His (%) | 6 (10.00) | 3 (5.00) | P<0.05 |
| Arg399Gln |  |  |  |
| Arg/Arg (%) | 38 (63.33) | 52 (86.67) | p>0.05 |
| Arg/Gln (%) | 14 (23.33) | 5 (8.33) | P<0.05 |
| Gln/Gln (%) | 8 (13.33) | 3 (5.00) | P<0.05 |

7. Han XC, Xing QZ, Li Y, Sun JJ, Ji HH, Pan HZ, et al. Study on the DNA repair gene XRCC1 and XRCC3 polymorphism in prediction and prognosis of hepatocellular carcinoma risk. [Hepato-Gastroenterol.](http://nc.yuntsg.com/one1.do) 2012; 59(119): 2285-2289. DOI: [10.5754/hge12096](http://doi.org/10.5754/hge12096).PMID: 22456434.

| Genotype | Liver cancer group (n=150) | Control group  (n=158) |
| --- | --- | --- |
| Arg194Trp |  |  |
| Arg/Arg (%) | 72 (47.8) | 84 (52.9) |
| Arg/Trp (%) | 47 (31.6) | 47 (29.5) |
| Trp/Trp (%) | 31 (20.6) | 28 (17.6) |
| Arg280His |  |  |
| Arg/Arg (%) | 82 (51.8) | 81 (53.9) |
| Arg/His (%) | 36 (22.7) | 35 (23.5) |
| His /His (%) | 40 (25.5) | 34 (22.6) |
| Arg399Gln |  |  |
| Arg/Arg (%) | 32 (21.33) | 46 (29.11) |
| Arg/Gln (%) | 78 (52.0) | 73 (46.20) |
| Gln/Gln (%) | 40 (26.67) | 1. 24.68) |

8. Yuan T, Deng SL, Liu HM, Liu MG, Chen P. Relationship between XRCC1 and XPD polymorphisms and the risk of the development of hepatocellular carcinoma: A case-control study. [Exp Ther Med.](http://nc.yuntsg.com/show.do?q=Relationship%20between%20XRCC1%20and%20XPD%20polymorphisms%20and%20the%20risk%20of%20the%20development%20of%20hepatocellular%20carcinoma:%20A%20case-control%20study.) 2012;4(2): 285-290. Epub 2012/05/17. DOI: [10.3892/etm.2012.581](http://doi.org/10.3892/etm.2012.581). PMID: 22970032.

| Genotype | HCC group (n=252) | Control group  (n=250) | OR (95% CI) | P-values |
| --- | --- | --- | --- | --- |
| Arg194Trp |  |  |  |  |
| Arg/Arg | 119 | 128 | 1.00 | 0.08 |
| Arg/Trp+ Trp/Trp | 133 | 122 | 1.56 (1.02-2.22) |  |
| Trp/Trp | 18 | 21 |  |  |
| Arg280His |  |  |  |  |
| Arg/Arg | 193 | 206 | 1.00 | 0.12 |
| Arg/His+ His /His | 59 | 44 | 1.43 (0.97-2.10) |  |
| His /His | 6 | 5 |  |  |

9. Zeng X, Liu S, Yu HP, Ji L, Li LM, Huang JM, Bai H, Qiu XQ. DNA repair capacity, DNA-strand break repair gene polymorphisms, and the incidence of hepatocellular carcinoma in southwestern Guangxi of China. [DNA Cell Biol.](http://nc.yuntsg.com/one1.do) 2012; 31(8): 1384-1391. Epub 2012/06/12. DOI: [10.1089/dna.2012.1646](http://doi.org/10.1089/dna.2012.1646). PMID: 22691054.

| Genotype | Cases (n = 46) | | | Controls (n = 46) | | |
| --- | --- | --- | --- | --- | --- | --- |
|  | n | Median of DRC | P | n | Median of DRC | P |
| rs1799782 |  |  |  |  |  |  |
| Arg/Arg | 23 | 0.80 | 0.72 | 26 | 0.96 | 0.42 |
| Arg/Trp+ Trp/Trp | 23 | 0.80 |  | 20 | 0.93 |  |
| rs25489 |  |  |  |  |  |  |
| Arg/Arg | 39 | 0.83 | 0.22 | 35 | 0.95 | 0.99 |
| Arg/His+ His /His | 7 | 0.76 |  | 11 | 0.96 |  |
| rs25487 |  |  |  |  |  |  |
| Arg/Arg | 33 | 0.80 | 0.71 | 25 | 0.93 | 0.55 |
| Arg/Gln+ Gln/Gln | 13 | 0.83 |  | 21 | 0.96 |  |

rs1799782: Arg194Trp; rs25489: Arg280His; rs25487: Arg399Gln.

10. Wu JS, Chen YP, Wang LC, Yang YJ, Deng CW, Hou BX, et al. Implication of polymorphisms in DNA repair genes with an increased risk of hepatocellular carcinoma. Genet Mol Res. 2014;13(2): 3812-3818. DOI: [10.4238/2014.May.16.5](http://doi.org/10.4238/2014.May.16.5). PMID:24938468

| Genotype | Cases (n=218) | % | Controls  (n=277) | % | P-values | OR (95% CI) |
| --- | --- | --- | --- | --- | --- | --- |
| Arg194Trp |  |  |  |  |  |  |
| Arg/Arg | 151 | 69.3 | 198 | 71.5 | - | 1.0 (Ref.) |
| Arg/Trp | 55 | 25.2 | 68 | 24.5 | 0.78 | 1.06 (0.68-1.64) |
| Trp/Trp | 12 | 5.5 | 11 | 4 | 0.40 | 1.43 (0.56-3.68) |
| Arg399Gln |  |  |  |  |  |  |
| Arg/Arg | 108 | 49.5 | 161 | 58.1 | - | 1.0 (Ref.) |
| Arg/Gln | 74 | 33.9 | 87 | 31.4 | 0.24 | 1.27 (0.84-1.92) |
| Gln/Gln | 36 | 16.6 | 29 | 10.5 | 0.02 | 1.85 (1.03-3.23) |

11. Yang ZH, Zhao J. Effect of APE1 and XRCC1 gene polymorphism on Susceptibility to hepatocellular carcinoma and sensitivity to cisplatin. Int J Clin Exp Med. 2015;8(6): 9931-9936. PMID: 26309678.

| Genotype | Liver cancer group (n=118) | Control group  (n=120) | OR (95% CI) | P |
| --- | --- | --- | --- | --- |
| Arg194Trp |  |  |  |  |
| Arg/Arg (%) | 55 (46.6) | 58 (48.3) | 1 |  |
| Arg/Trp (%) | 53 (44.9) | 45 (37.5) | 1.242 (0.722~2.136) | 0.433 |
| Trp/Trp (%) | 10 (8.5) | 17 (14.2) | 0.620 (0.261~1.471) | 0.279 |

12. Krupa R, Czarny P, Wigner P, Wozny J, Jablkowski M, Kordek R, et al. The Relationship Between Single-Nucleotide Polymorphisms, the Expression of DNA Damage Response Genes, and Hepatocellular Carcinoma in a Polish Population. DNA Cell Biol.2017;36(8): 693-708. Epub 2017/06/09.DOI: 10.1089/dna.2017.3664. PMID:28598207.

| Genotype | HCC group (n=65) | Control group  (n=50) | Crude OR (95% CI) | P-values |
| --- | --- | --- | --- | --- |
| Arg194Trp |  |  |  |  |
| Arg/Arg | 57 (0.877) | 41 (0.820) | 1.56 (0.56-4.40) | 0.396 |
| Arg/Trp | 5 (0.077) | 8 (0.160) | 0.44(0.13-1.43) | 0.171 |
| Trp/Trp | 3 (0.046) | 1 (0.020) | 2.37 (0.24-23.51) | 0.461 |
| Arg280His |  |  |  |  |
| Arg/Arg | 57 (0.877) | 36 (0.720) | 2.77 (1.06-7.23) | 0.038 |
| Arg/His | 7 (0.108) | 11 (0.220) | 0.43 (0.15-1.20) | 0.107 |
| His /His | 1 (0.015) | 3 (0.060) | 0.24 (0.02-2.43) | 0.229 |
| Arg399Gln |  |  |  |  |
| Arg/Arg | 42 (0.646) | 32 (0.640) | 1.03 (0.48-2.22) | 0.946 |
| Arg/Gln | 15 (0.231) | 12 (0.240) | 0.95 (0.40-2.26) | 0.908 |
| Gln/Gln | 8 (0.123) | 6 (0.120) | 1.03 (0.33-3.18) | 0.960 |

13. Guo LY, Jin XP, Niu W, Li XF, Liu BH, Wang YL. Association of XPD and XRCC1 Genetic Polymorphisms with Hepatocellular Carcinoma Risk. Asian Pac J Cancer Prev. 2012; 13(9): 4423-4426. PMID: 23167354.

| Genotype | Cases (n=410) | % | Controls (n=410) | % | P-values | OR (95% CI) |
| --- | --- | --- | --- | --- | --- | --- |
| Arg194Trp |  |  |  |  |  |  |
| Arg/Arg | 264 | 64.4 | 292 | 71.2 | 0.22 | 1.0 (Ref.) |
| Arg/Trp | 109 | 26.5 | 96 | 23.3 |  | 1.17(0.83-1.55) |
| Trp/Trp | 37 | 9.1 | 23 | 5.5 |  | 2.26(1.23-5.38) |
| Arg399Gln |  |  |  |  |  |  |
| Arg/Arg | 203 | 49.6 | 227 | 55.3 | 0.17 | 1.0 (Ref.) |
| Arg/Gln | 136 | 33.1 | 128 | 31.3 |  | 1.16(0.86-1.62) |
| Gln/Gln | 71 | 17.3 | 55 | 13.4 |  | 1.74(1.06-2.74) |

14. Wu H, Yang Z, Xie Y, Kuang Z, Luo X, Liang A, et al. Correlation between DNA repair gene XRCC1 Arg280His polymorphism and susceptibility to hepatocellular carcinoma in Fusui county of Guangxi. China Journal of Modern Medicine. 2009; 19(18): 2737-2743.

|  | Zhuang | | | Han | | |
| --- | --- | --- | --- | --- | --- | --- |
| Genotype | Cases (n=50) | Controls  (n=30) | OR (95% CI) | Cases (n=50) | Controls  (n=30) | OR (95% CI) |
| Arg280His |  |  |  |  |  |  |
| Arg/Arg | 40 (80) | 24 (80) | 1.00^ref^ | 37 (74) | 23 (76.7) | 1.00^ref^ |
| Arg/His | 9 (18) | 6 (20) | 0.900(0.285- 2.843) | 13 (26) | 7 (23.3) | 1.154(0.402- 3.319) |
| His/His | 1 (2) | 0 |  | 0 | 0 |  |

15. Yuan T, Wei JY, Luo J, Liu MG, Deng SL, Chen P. Polymorphisms of base-excision repair genes hOGG1 326cys and XRCC1 280His increase hepatocellular carcinoma risk. Dig Dis Sci. 2012; 57(9): 2451-2457. Epub 2012/05/08. DOI: 10.1007/s10620-012-2192-6. PMID:22565339.

| Arg280His | Cases(%) | Controls(%) | OR | 95% CI | P value |
| --- | --- | --- | --- | --- | --- |
| Arg/Arg | 272 (77.7) | 329 (82.3) | 1.00 |  |  |
| Arg/His | 73 (20.9) | 64 (15.9) | 1.38 | 0.95–2.00 | 0.107 |
| His /His | 5 (1.4 %) | 7 (1.75 %) | 0.91 | 0.28–2.81 | 1.000 |

16. Bo WL, Zhang GX, Li DY, Wang X. [Study of polymorphisms of DNA repair gene XRCC1 and hepatic cancer](http://g.wanfangdata.com.cn/details/detail.do?_type=perio&id=hebykdxxb201201011). Harbin Med J. 2012; 01:43-45. DOI:[10.3969/j.issn.1000-1905.2012.01.011](http://g.wanfangdata.com.cn/details/javascript:void(0);).

| Genotype | Cases(%) n=90 | Controls(%) n=90 | P |
| --- | --- | --- | --- |
| Arg280His |  |  |  |
| Arg/Arg | 64 (71. 11) | 78 (86. 67) | >0. 05 |
| Arg/His | 18 (20. 00) | 9 (10. 00) | <0. 05 |
| His /His | 8 (8. 89) | 3 (3. 33) | <0. 05 |

| Arg280His | Arg/Arg (%) | Arg/His (%) | His/His (%) | His allele frequency | Arg allele frequency | Significant test |
| --- | --- | --- | --- | --- | --- | --- |
| Control (n=74) | 44 (59) | 27 (36) | 3 (4) | 0.22 | 0.78 | X2=9.28, d.f.=4, P=0.06 |
| HCC patients (n=50) | 24 (48) | 17 (34) | 9 (18) | 0.35 | 0.65 |  |
| Arg399Gln | Arg/Arg | Arg/Gln | Gln/Gln | Gln allele frequency | Arg allele frequency | Significant test |
| Control (n=74) | 27 (36) | 32 (43) | 15 (20) | 0.42 | 0.58 | X2=12.03, d.f.=4, P=0.02 |
| HCC patients (n=50) | 19 (38) | 14 (28) | 17 (34) | 0.48 | 0.52 |  |

17. Gulnaz A, Sayyed AH, Amin F, Khan Au, Aslam MA, Shaikh RS, et al. Association of XRCC1, XRCC3, and XPD genetic polymorphism with an increased risk of hepatocellular carcinoma because of the hepatitis B and C virus. Eur J Gastroenterol Hepatol. 2013; 25(2): 166-179. DOI:10.1097/MEG.0b013e328359a775. PMID:23044807.

18. He CC, Xie YA, Zhao RQ, Yan L. The relationship of XRCC1 Arg 399GLn (rs25487) and Arg280His (rs25489) polymorphisms with the family genetic susceptibility to hepatocellular carcinoma of Zhuang population in Fusui county of Guangxi.Chin J Cancer Biother. 2015; 22(3): 348-353.

| Genotype | Genotype HCC high incidence families ( N = 79) | χ2 | P | Normal control families ( N = 40) | χ2 | P |
| --- | --- | --- | --- | --- | --- | --- |
| Arg280His |  |  |  |  |  |  |
| Arg/Arg | 47 | 0.03 | 0.87 | 27 | 0.06 | 0.80 |
| Arg/His | 26 |  |  | 12 |  |  |
| His /His | 4 |  |  | 1 |  |  |
| Arg399Gln |  |  |  |  |  |  |
| Arg/Arg | 61 | 1.04 | 0.31 | 26 | 0.11 | 0.74 |
| Arg/Gln | 16 |  |  | 4 |  |  |
| Gln/Gln | 0 |  |  | 0 |  |  |

19. Yao JG, Huang XY, Long XD. Interaction of DNA repair gene polymorphisms and aflatoxin B1 in the risk of hepatocellular carcinoma. Int J Clin Exp Pathol. 2014;7(9):6231-6244. PMID: 25337275.Int J Clin Exp Pathol.

| Arg399Gln | Cases (%) | Controls (%) | OR | 95% CI | P |
| --- | --- | --- | --- | --- | --- |
| Arg/Arg | 777 (52.29) | 1437 (71.99) | Reference |  |  |
| Arg/Gln | 608 (40.92) | 520 (26.05) | 2.155 | 1.861-2.495 | 9.918 × 10^-25^ |
| Gln/Gln | 101 (6.80) | 39 (1.95) | 4.774 | 3.264-6.981 | 7.614 × 10^-16^ |

| Arg399Gln | Arg/Arg | Arg/Gln | Gln/Gln | P_trend_ |
| --- | --- | --- | --- | --- |
| Cases | 301 (52.2) | 223 (38.6) | 53 (9.2) |  |
| Controls | 218 (56.0) | 143 (36.8) | 28 (7.2) |  |
| Univariate OR (95% CI) | 1.00 (referent) | 1.13 (0.86 to 1.48) | 1.37 (0.84 to 2.24) | .166 |
| Multivariable OR (95% CI) | 1.00 (referent) | 1.10 (0.82 to 1.46) | 1.54 (0.92 to 2.58) | .129 |

20. Yu MW, Yang SY, Pan IJ, Lin CL, Liu CJ, Liaw YF, et al. Polymorphisms in XRCC1 and glutathione S-transferase genes and hepatitis B-related hepatocellular carcinoma. J Natl Cancer Inst.2003; 95 (19): 1485-1488. PMID:14519756.

21. Yang JL, Han YN, Zhen SG. Influence of human XRCC1-399 single nucleotide polymorphism on primary hepatocytic carcinoma. Tumor. 2004; 24(4): 322-324.

| Arg399Gln | Cases (%) | Controls (%) | P value |
| --- | --- | --- | --- |
| Arg/Arg | 34 (16.6) | 58 (28.3) |  |
| Arg/Gln | 7 (3.4) | 15 (7.3) |  |
| Gln/Gln | 28 (13.7) | 63 (30.7) | 0.663 |

22. Long XD, Ma Y, Wei YP, Deng ZL. Polymorphism of DNA repair gene XRCC1 and risk of hepatocellular carcinoma. J Guangxi Med Univ. 2004; 21(3): 313-315.

| Arg399Gln | Cases (%) | Controls (%) | adjusted OR |
| --- | --- | --- | --- |
| Arg/Arg | 72 (51.43) | 362 (67.54) | 1^ref^ |
| Arg/Gln | 63 (45.00) | 159 (29.66) | 2.08 (1.20-3.62) |
| Gln/Gln | 5 (3.57) | 15 (2.80) | 4.02 (0.93-17.45) |

23. Kirk GD, Turner PC, Gong Y, Lesi OA, Mendy M, Goedert JJ, et al. Hepatocellular carcinoma and polymorphisms in carcinogen-metabolizing and DNA repair enzymes in a population with aflatoxin exposure and hepatitis B virus endemicity.Cancer Epidemiol Biomarkers Prev. 2005; 14 (2): 373-379. DOI: 10.1158/1055-9965.EPI-04-0161. PMID: 15734960.

| Arg399Gln | Cases (%) | Controls (%) |
| --- | --- | --- |
| Arg/Arg | 120 (80.5) | 248 (84.4) |
| Arg/Gln | 26 (17.5) | 43 (14.6) |
| Gln/Gln | 3 (2.0) | 3 (1.0) |

24. Borentain P, Gerolami V, Ananian P, Garcia S, Noundou A, Botta-Fridlund D, et al. DNA-repair and carcinogen-metabolising enzymes genetic polymorphisms as an independent risk factor for hepatocellular carcinoma in Caucasian liver-transplanted patients. [Eur J Cancer.](http://nc.yuntsg.com/one1.do) 2007; 43(17): 2479-2486. Epub 2007/09/17. DOI: 10.1016/j.ejca.2007.08.006. PMID: 17870518.

| Arg399Gln | Transplanted patients (n=133) | | Odds Ratio (95% CI) HCC versus non HCC | Healthy subjects (n=89) | Odds Ratio (95% CI) HCC versus healthy subjects |
| --- | --- | --- | --- | --- | --- |
|  | HCC (n=56) | non HCC (n=77) |  |  |  |
| Arg/Arg | 27 (48%) | 29 (38%) | 1.54 (0.72–3.29) | 27 (30%) | 2.30 (1.01–4.53) p=0.03 |
| Arg/Gln | 21 (37.5%) | 35 (45%) | 0.72 (0.34–1.54) | 43 (48%) | 0.64 (0.31–1.34) |
| Gln/Gln | 8 (15%) | 13 (17%) | 0.82 (0.28–2.34) | 19 (21%) | 0.61 (0.23–1.64) |

25. Ren Y, Wang DS, Li Z, Xin YM, Yin JM, Zhang B, et al. Study on the Relationship between Gene XRCC1 Codon 399 Single Nucleotide Polymorphisms and Primary Hepatic Carcinoma in Han Nationality. Linchuang Ganzangbing Zazhi. 2008; 24(5): 361-364.

| Arg399Gln | Cases (%) | Controls (%) |
| --- | --- | --- |
| Arg/Arg | 32 (64.0) | 46 (50.0) |
| Arg/Gln | 14 (28.0) | 41 (44.5) |
| Gln/Gln | 4 (8.0) | 5 (5.5) |

26. Jia ZF, Su HY, Li XL, Xu X, Yin ZH, Guan P, et al. Polymorphisms of UGT1A7 and XRCC1 are associated with an increased risk of hepatocellular carcinoma in Northeast China. Chin J Cancer Res. 2010;22(4): 260-266. DOI: 10.1007/s11670-010-0260-z.

| Arg399Gln | HCC (n=136) | Controls (n=136) | OR(95% CI) | P |
| --- | --- | --- | --- | --- |
| Arg/Arg | 53 | 78 | 1.00(reference) | — |
| Arg/Gln | 66 | 45 | 2.16(1.29-3.61) | 0.003 |
| Gln/Gln | 17 | 13 | 1.93(0.86-4.29) | 0.108 |
| Allele frequencies |  |  |  |  |
| Arg | 172 | 201 | 1.65(1.14-2.73) | 0.007 |
| Gln | 100 |  |  |  |

27. Pan HZ, Liang J, Yu Z, Lun LM, Li H,Wang Q. Polymorphism of DNA Repair Gene XRCC1 and Hepatocellular Carcinoma Risk in Chinese Population. Asian Pac J Cancer Prev. 2011; 12: 2947-2950. PMID: 22393969.

| Arg399Gln | Cases (%) | Controls (%) |
| --- | --- | --- |
| Arg/Arg | 45 (22.28) | 68 (28.81) |
| Arg/Gln | 105 (51.98) | 112 (47.46) |
| Gln/Gln | 52 (25.74) | 56 (23.73) |

28. He GZ, Huang TR, Deng W, Yu JH, Zhang CY, Li JL. Research on Association Between XRCC1Arg399Gln Polymorphism and Liver Cancer. J Guangxi Univ Chin Med. 2012; 15(3): 1-3.

| Arg399Gln | Cases (n=113) | Controls (n=113) | P |
| --- | --- | --- | --- |
| Arg/Arg | 80 (70.79) | 97 (85.84) | >0. 05 |
| Arg/Gln | 23 (20.35) | 12 (10.61) | <0. 05 |
| Gln/Gln | 10 (8.85) | 4 (3.54) | <0. 05 |

29. Mohana DS, Balachandar V, Arun M, Suresh KS, Balamurali KB, Sasikala K. Analysis of genetic damage and gene polymorphism in hepatocellular carcinoma (HCC) patients in a South Indian population. Dig Dis Sci. 2013;58(3): 759-767. DOI:10.1007/s10620-012-2409-8.

| Arg399Gln | HCC patients (n=93) | Controls (n=93) | OR(95%CI) | P |
| --- | --- | --- | --- | --- |
| Arg/Arg | 36 (38.71) | 32 (34.41) | 1 (Ref.) | 0.234 |
| Arg/Gln | 45 (48.39) | 51 (54.84) | 0.78 (0.42–1.46) |  |
| Gln/Gln | 12 (12.90) | 10 (10.75) | 1.36 (0.54–3.45) |  |

30. Bose S, Tripathi DM, Sukriti, Sakhuja P, Kazim SN, Sarin SK. Genetic polymorphisms of CYP2E1 and DNA repair genes HOGG1 and XRCC1: association with hepatitis B related advanced liver disease and cancer. Gene. 2013; 519(2): 231-237. Epub 2013/02/27. DOI: 10.1016/j. gene.2013.02.025. PMID: 23454624.

| Arg399Gln | HCC patients (n=55) | Controls (n=210) |
| --- | --- | --- |
| Arg/Arg | 22 | 75 |
| Arg/Gln | 29 | 88 |
| Gln/Gln | 4 | 46 |

31. Santonocito C, Scapaticci M, Nedovic B, Annicchiarico EB, Guarino D, Leoncini E, et al. XRCC1 Arg399Gln gene polymorphism and hepatocellular carcinoma risk in the Italian population. Int J Biol Markers 2017; 32(2): e190-e194. DOI: 10.5301/jbm.5000241.

| Arg399Gln | HCC patients (n=89) | Controls (n=99) | OR (95% CI) | OR (95% CI)* | OR (95% CI)† |
| --- | --- | --- | --- | --- | --- |
| Arg/Arg | 37 (41.57) | 59 (59.60) | 1.0 (reference) | 1.0 (reference) | 1.0 (reference) |
| Arg/Gln | 45 (50.56) | 38 (38.38) | 1.88 (1.04-3.43) | 1.94 (1.04-3.63) | 1.66 (0.51-5.34) |
| Gln/Gln | 7 (7.87) | 2 (2.02) | 2.34 (0.98-7.54) | nc | nc |

32. Bazgir A, Gholizadeh MA, Khosravi A, Samaei NM. The X-ray Repair Cross-Complementing Group 1 Arg399Gln Genetic Polymorphism and Risk of Hepatocellular Carcinoma in an Iranian Population. Middle East J Dig Dis. 2017;10(1):40-44. DOI: 10.15171/mejdd.2017.89.

| Arg399Gln | Cases (n=50) | Controls (n=101) |
| --- | --- | --- |
| Arg/Arg | 12 | 31 |
| Arg/Gln | 18 | 56 |
| Gln/Gln | 20 | 14 |
