# Supplementary material for: Associations between three XRCC1 polymorphisms and hepatocellular carcinoma risk: A meta-analysis of case-control studies
Source: PLoS One. 2018 Nov 8;13(11):e0206853. doi: 10.1371/journal.pone.0206853 (PMC6226104; doi:10.1371/journal.pone.0206853)
Supplement: S1 Appendix — (DOC) [file pone.0206853.s006.doc]

Reasons for excluded studies

**1.Case only studies:**

1. Guan Q,Chen Z,Chen Q.XRCC1 and XPD polymorphisms and their relation to the clinical course in hepatocarcinoma patients.Oncol Lett 2017;14:2783-2788.DOI:10.3892/ol.2017.6522
2. Yue AM,Xie ZB,Guo SP,et al.Implication of polymorphisms in DNA repair genes in prognosis of hepatocellular carcinoma.Asian Pac J Cancer Prev 2013;14:355-358.
3. Zeng XY, Huang JM, Xu Y, et al. [Interactions between XRCC1 gene polymorphism and specific environmental factors in HCC cases](http://g.wanfangdata.com.cn/details/detail.do?_type=perio&id=qlzlzz201217001). Chin J Cancer Prev & Trea. 2012;17:1281-1284. DOI: 10.16073/j.cnki.cjcpt.2012.17.002.

**2. reviews**

1. Ye XP, Peng T, Li LQ. Study on polymorphisms in metabolic enzyme genes, DNA repair genes and individual susceptibility to hepatocellular carcinoma. J Hygiene Res. 2006;06:805-807. DOI:[10.3969/j.issn.1000-8020.2006.06.043](http://g.wanfangdata.com.cn/details/javascript:void(0);).
2. Huang JM, Zeng XY. DNA repair gene XRCC1 single nucleotide polymorphism and liver cancer susceptibility. Preventive Medicine Tribune. 2010;16(05):448-451. DOI: 10.16406/j.pmt.issn. 1672-9153.2010.05.042

**3.Meta-analysis:**

1. Li W,Yang F,Gui Y. DNA repair gene XRCC1 Arg194Trp polymorphism and susceptibility to hepatocellular carcinoma: A meta-analysis.Oncol Lett 2014;8:1725-1730. DOI:10.3892/ol.2014.2351.
2. Pan Y,Zhao L,Chen XM,et al.The XRCC1 Arg399Gln genetic polymorphism contributes to hepatocellular carcinoma susceptibility: an updated meta-analysis.Asian Pac J Cancer Prev 2013;14:5761-5767.
3. Qi Y,Cui L,Song Y. XRCC1 Arg399Gln genetic polymorphism and the risk of hepatocellular carcinoma: a meta-analysis.Mol Biol Rep 2014;41:879-887.DOI:10.1007/s11033-013-2929-0
4. Shi YH,Wang B,Xu BP,et al.The association of six non-synonymous variants in three DNA repair genes with hepatocellular carcinoma risk: a meta-analysis.J Cell Mol Med 2016;20:2056-2063.DOI:10.1111/jcmm.12896
5. Wang YD,Zhai WL,Wang HY. An updated meta-analysis on the association of X-ray repair cross complementing group 1 codon 399 polymorphism with hepatocellular carcinoma risk.Asian Pac J Cancer Prev 2014;15:4443-4448.
6. Xu W,Liu SA,Li L,et al. Association between XRCC1 Arg280His polymorphism and risk of hepatocellular carcinoma: a systematic review and meta-analysis.Genet Mol Res 2015;14:7122-7129.DOI:10.4238/2015.June.29.5
7. Zhang XL,Lu Y,Yang S,et al. An updated meta-analysis between the association of XRCC1 Arg399Gln polymorphism and hepatocellular carcinoma risk.Asian Pac J Cancer Prev 2014;15:3273-3278.
8. Li J,Li Z,Feng L,et al.Polymorphisms of DNA repair gene XRCC1 and hepatocellular carcinoma risk among East Asians: a meta-analysis.Tumour Biol 2013;34:261-269.DOI:10.1007/s13277-012-0546-5
9. Li LP,Wu W,Li XH.The XRCC1 Arg280His gene polymorphism and hepatocellular carcinoma risk: a meta-analysis.Asian Pac J Cancer Prev 2013;14:2033-2036.
10. Liu F,Li B,Wei Y,et al.XRCC1 genetic polymorphism Arg399Gln and hepatocellular carcinoma risk: a meta-analysis.Liver Int 2011;31:802-809.DOI:10.1111/j.1478-3231.2011.02508.x
11. Wu D,Jiang H,Gu Q,et al.Association between XRCC1 Arg399Gln polymorphism and hepatitis virus-related hepatocellular carcinoma risk in Asian population.Tumour Biol 2013;34:3265-3269.DOI:10.1007/s13277-013-0899-4
12. Xie T,Wang ZG,Zhang JL.X-ray repair cross-complementing group 1 polymorphisms and hepatocellular carcinoma: a meta-analysis.World J Gastroenterol 2012;18:4207-4214. DOI:10.3748/wjg.v18.i31.4207
13. Zeng XY, Huang JM, Xu JW, et al .Meta-analysis demonstrates lack of a relationship between XRCC1-399 gene polymorphisms and susceptibility to hepatocellular carcinoma.Genet Mol Res 2013;12:1916-1923.DOI:10.4238/2013.March.15.5
14. Zhang H,Wang P,Dai L,et al Meta-analysis of the relationship between polymorphisms of 399 point of X-ray repair cross complementing gene group 1 and hepatocellular carcinoma.Wei Sheng Yan Jiu 2010;39:664-668.
15. Li J,Li Z,Feng L,et al.Polymorphisms of DNA repair gene XRCC1 and hepatocellular carcinoma risk among East Asians: a meta-analysis.Tumour Biol 2013;34:261-269.DOI:10.1007/s13277-012- 0546-5.
16. Yi L,Xiao-Feng H,Yun-Tao L,et al.Association between the XRCC1 Arg399Gln polymorphism and risk of cancer: evidence from 297 case-control studies.PLoS One 2013;8:e78071.DOI:10.1371/journal.pone.0078071
17. Qi Y,Cui L,Song Y.XRCC1 Arg399Gln genetic polymorphism and the risk of hepatocellular carcinoma: a meta-analysis.Mol Biol Rep 2014;41:879-887.DOI:10.1007/s11033-013-2929-0
18. Liu J, Zhu QM, Hu HY, Wang S. Association between XRCC1 Arg399Gln polymorphism and susceptibility to hepatocellular carcinoma in Chinese populations: A Meta-analysis. World Chin J Gastroenterol. 2015; 23(15): 2468-2474. DOI: 10.11569/wcjd.v23.i15.2468.
19. Li SH, Li Y, Ma JZ. Association analysis of XRCC1 genetic polymorphism loci with primary heptocellular carcinoma in chinese population. [Prog Anatom Sci](http://g.wanfangdata.com.cn/details/javascript:void(0)).[2012, 2](http://g.wanfangdata.com.cn/details/javascript:void(0)):147-150.

http://g.wanfangdata.com.cn/details/detail.do?_type=degree&id=Y2226862.

1. Duan WH,Zhu ZY,Liu JG,et al. XRCC1 Arg399Gln gene polymorphism and hepatocellular carcinoma risk in the Chinese Han population: a meta-analysis.Asian Pac J Cancer Prev 2012;13:3601-3604.

**4.Data duplication**

1. Li QW,Lu CR,Ye M,et al.Evaluation of DNA repair gene XRCC1 polymorphism in prediction and prognosis of hepatocellular carcinoma risk.Asian Pac J Cancer Prev 2012;13:191-194.
2. Xu L. Case-control Study of DNA Repair Gene Polymorphism and Susceptibility to Primary Hepatocellular Carcinoma. Fudan University. 2003. DOI:[10.7666/d.y556061](http://g.wanfangdata.com.cn/details/javascript:void(0);).
3. Han YN, Yang JL, Zhen SY, Hu YQ. Study on the association of human XRCC1-399 single nucleotide polymorphism and primary hepatocytic carcinoma. Hepatology. 2004;9(04):235-237.
4. Chen CC, Yang SY, Liu CJ, Lin CL, Liaw YF, Lin SM, et al. Association of cytokine and DNA repair gene polymorphisms with hepatitis B-related hepatocellular carcinoma. Int J Epidemiol. 2005;34(6): 1310-1318. Epub 2005/09/19. DOI: 10.1093/ije/dyi191. PMID: 16172101.

**5.Unable to attach full text**

1. Avadanei R, Amalinei C, Giusca S, Grigoras A, Caruntu ID. Polymorphism of DNA repair genes XRCC1 and XRCC3 and hepatocellular carcinoma risk in Romanian population. Virchows Archiv. 2015;467:S227-S227.
