# Supplementary material for: Associations between three XRCC1 polymorphisms and hepatocellular carcinoma risk: A meta-analysis of case-control studies
Source: PLoS One. 2018 Nov 8;13(11):e0206853. doi: 10.1371/journal.pone.0206853 (PMC6226104; doi:10.1371/journal.pone.0206853)
Supplement: S2 Appendix — (DOCX) [file pone.0206853.s007.docx]

The differences from the previous meta-analyses.

Background: XRCC1 gene polymorphism is still a hot topic in cancer research. There is no genome-wide association study (GWAS) relevant to XRCC1 gene polymorphisms of hepatocellular carcinoma (HCC). However, many meta-analyses about XRCC1 gene polymorphism were reported, but conclusions are inconsistent. This research endeavored to collect relevant literature, hoping that a reliable conclusion can be reached to end the controversy.

Conclusion of our meta-analysis

A total of 32 case-control studies, including 13 that evaluated Arg194Trp, 14 that evaluated Arg280His, and 26 that evaluated Arg399Gln, were analyzed. In the entire study population, XRCC1 Arg399Gln was significantly associated not only with overall risk of HCC (homozygous model, OR = 1.61, 95% CI: 1.40–1.85, P < 0.05; recessive model, OR = 1.40, 95% CI: 1.23–1.59, P < 0.05) but also with the risk of HCC in Chinese patients (homozygous model, OR = 1.78, 95% CI: 1.53–2.08, P < 0.05; recessive model, OR = 1.47, 95% CI: 1.27–1.70, P < 0.05). Limiting the analysis to studies demonstrating Hardy–Weinberg equilibrium (HWE), the results were consistent and robust. Similarly, a significant association between XRCC1 Arg399Gln and HCC risk was found in healthy controls in the general population but not in hospital controls. Trial sequential analysis (TSA), false-positive report probabilities (FPRP), and combined genotype analysis revealed that XRCC1 Arg399Gln is mainly associated with susceptibility to liver cancer. However, there was no association between Arg194Trp or Arg280His and the risk of HCC. These results, indicating that the Arg399Gln polymorphism of XRCC1 is associated with the risk of HCC in the Chinese population, provide a basis for the development of improved detection and treatment approaches.

Conclusion of previous studies and differences between us:

① (Li et al. 2012) The Arg399Gln polymorphisms of XRCC1 may be a genetic susceptibility for HCC in the East Asian population (Gln/Gln vs. Arg/Arg, OR=1.32, 95% CI=1.08-1.61; Gln/Gln + Arg/Gln vs. Arg/Arg, OR=1.39, 95%CI =01.15-1.69). Limiting the analysis to the studies within Hardy-Weinberg equilibrium, the results were persistent and robust. But no evidence of association of Arg194Trp and Arg280His with HCC risk was found.

Studies included in the meta-analysis (n=15), For XRCC1 Arg399Gln polymorphism (n=13) , Arg194Trp polymorphism (n=5), Arg280His polymorphism (n=5).

Reference:

Li J, Li ZZ, Feng LS, Guo WZ, Zhang SJ. Polymorphisms of DNA repair gene XRCC1 and hepatocellular carcinoma risk among East Asians: a meta-analysis. Tumor Biol. 2013; 34: 261-269. DOI:10.1007/s13277-012-0546-5.

Differences:

Firstly, our meta-analysis included more researches, for XRCC1 Arg399Gln polymorphism (n=26), Arg194Trp polymorphism (n=13), Arg280His polymorphism (n=14). Secondly, Li et al. believed that XRCC1 Arg280His polymorphisms were not related to the susceptibility of hepatocellular carcinoma. Besides, our conclusion is consistent with Li et al., but we calculated the false positive rate and performed the TSA, which proved that Arg399Gln was significantly associated with liver cancer risk. Our conclusions are more robust.

② (Shi et al. 2016) Arg280His associated with the significant risk for hepatocellular carcinoma (Allelic model, OR=1.37, 95%CI=1.13-1.66, P=0.001; Dominant model, OR=1.53, 95%CI=1.11-2.10, P=0.010). XRCC1 gene Arg399Gln might be candidate susceptibility loci for hepatocellular carcinoma (Allelic model, OR=1.19, 95%CI= 0.97-1.46, P=0.093; Dominant model, OR=1.24, 95%CI= 0.97-1.58, P=0.082). Subgroup analyses revealed significant association of XRCC1 gene Arg399Gln with hepatocellular carcinoma in Chinese especially from south China (OR=1.57, 95%CI= 1.16-2.14, P=0.004).

Studies included in the meta-analysis, For XRCC1 Arg399Gln polymorphism (n=13), Arg280His polymorphism (n=4).

Reference:

Shi YH, Wang B, Xu BP, Jiang DN, Zhao DM, Ji MR et al. The association of six non-synonymous variants in three DNA repair genes with hepatocellular carcinoma risk: a meta-analysis. J Cell Mol Med. 2016; 20(11):2056-2063. Epub 2016/06/16. DOI: 10.1111/jcmm. 12896. PMID:27306318.

Differences:

Firstly, our meta-analysis included more researches, for XRCC1 Arg399Gln polymorphism (n=26), Arg194Trp polymorphism (n=13), Arg280His polymorphism (n=14). Secondly, the conclusion that XRCC1 geneArg280His and Arg399Gln polymorphisms were related to risk of hepatocellular carcinoma was not persuasive enough, because it was only based on allelic model and dominant model. However, our meta-analysis was based on four models (including homozygous genetic model, heterozygous genetic model, dominant genetic model and recessive genetic model), which were widely used by many scholars, and thus the conclusion would be more accurate.

③ (Xie et al. 2012）No associations between the Arg194Trp and Arg399Gln polymorphisms of the XRCC1 gene and HCC risk were found. And His/His genotype of Arg280His polymorphism had a significant association with the increased risk of HCC (His/His vs. Arg/Arg, OR=1.96, 95%CI=1.03-3.75, P=0.04). However, the sensitivity analysis showed that there is no association.

Studies included in the meta-analysis (n=13), For XRCC1 Arg399Gln polymorphism (n=9) , Arg194Trp polymorphism (n=4), Arg280His polymorphism (n=4).

Reference:

Xie T, Wang ZG, Zhang JL.X-ray repair cross-complementing group 1 polymorphisms and hepatocellular carcinoma: a meta-analysis. World J Gastroenterol 2012;18:4207-4214.DOI:10.3748/wjg.v18.i31.4207.

Differences:

Firstly, our meta-analysis included more researches, for XRCC1 Arg399Gln polymorphism (n=26), Arg194Trp polymorphism (n=13), Arg280His polymorphism (n=14). Secondly, Xie et al. considered that XRCC1 gene Arg399Glnpolymorphisms ware not associated with the risk of hepatocellular carcinoma, which is opposite to our conclusion. Thirdly, they thought His/His genotype of Arg280His polymorphism was significantly associated with the increased risk of hepatocellular carcinoma (His/His vs. Arg/Arg, OR=1.96, 95%CI=1.03-3.75, P=0.04). In our mate-analysis, we found that Arg280His polymorphisms was not associated with the susceptibility of hepatocellular carcinoma (homozygous model, OR = 1.43, 95% CI: 0.91–2.25, P= 0.15; recessive model, OR = 1.15, 95% CI: 0.84–1.56, P = 0.22).

④(Liu et al) No association is found between the XRCC1 polymorphisms Arg399Gln and the risk of HCC. When stratifying for ethnicity, country/region and source of controls, no evidence of a significant association was observed in any subgroup.

Studies included in the meta-analysis for XRCC1 Arg399Gln polymorphism (n=11).

Reference:

Liu F, Li B, Wei Y, et al. XRCC1 genetic polymorphism Arg399Gln and hepatocellular carcinoma risk: a meta-analysis. Liver Int 2011;31:802-809.

DOI:10.1111/j.1478-3231.2011.02508.x.

Differences:

Firstly, the conclusion of our meta-analysis is contrary to that of Liu et al. They considered that XRCC1 gene Arg399Gln polymorphisms were not related to the risk of hepatocellular carcinoma. While in our mate-analysis, we found a significant correlation between them. Limiting the analysis to the studies within HWE, the results were persistent and robust. Secondly, the literature we included was far more than Liu et al.’s (n=26 vs. n=11), which means a better accuracy and a stronger persuasiveness.

⑤(Wang et al)XRCC1 gene Arg399Gln polymorphisms were significantly associated with the susceptibility of hepatocellular carcinoma in Chinese people(G/G vs. A/A, OR=1. 32, 95%CI=1. 13-1. 54，P=0. 000; G/G vs. A/A + A/G, OR =1. 20, 95%CI:=1. 04-1. 39，P =0. 014). Subgroup analysis based on regional grouping, there was a significant association between XRCC1 Arg399Gln polymorphisms and the susceptibility of hepatocellular carcinoma (G/G vs. A/A, OR = 1. 47，95% CI:1. 10-1. 95，P=0. 009; A/G vs. A/A, OR=1. 35, 95%CI:1. 17-1. 56, P =0. 000; A/G + G/G vs. A/A, OR= 1. 33, 95% CI:1. 16-1. 52, P=0. 000).

Studies included in the meta-analysis for XRCC1 Arg399Gln polymorphism (n=17).

Reference:

Wang Q, Tan SY. An updated meta-analysis of the association between XRCC1 Arg399Gln polymorphism and hepatocellular carcinoma risk in Chinese population. [Chin Clin Oncol](http://g.wanfangdata.com.cn/details/javascript:void(0)).2017;(4):319-325. DOI:10.3969/j.issn.1009-0460.2017.04.007.

Differences:

Firstly, our meta-analysis included more researches, for XRCC1 Arg399Gln polymorphism (n=26). Secondly, wang et al. did not restrict the analysis to HWE research, so its results might not be stable. In our meta-analysis, XRCC1 Arg399Gln was significantly associated not only with the overall risk of HCC (homozygous model, OR = 1.61, 95% CI: 1.40–1.85, P < 0.05; recessive model, OR = 1.40, 95% CI:1.23–1.59, P < 0.05), but also with the risk of HCC in Chinese patients (homozygous model, OR = 1.78, 95% CI: 1.53–2.08, P < 0.05; recessive model, OR = 1.47, 95% CI: 1.27–1.70, P < 0.05). Limiting the analysis to studies demonstrating HWE, the results were consistent and robust. Similarly, a significant association between XRCC1 Arg399Gln and HCC risk was found in healthy controls in the general population but not in hospital controls. TSA, FPRP and the combined genotype analysis revealed that XRCC1 Arg399Gln is mainly associated with susceptibility to liver cancer. Therefore, our conclusion is more reliable.

⑥(Liu et al )XRCC1 Arg399Gln polymorphism seems to be involved in hepatocellular carcinoma risk. XRCC1 399Gln/Gln genotype increases the susceptibility to hepatocellular carcinoma in Chinese populations (Arg/Arg vs. Gln/Gln, OR = 1.47, 95%CI: 1.24-1.74, P < 0.001; Gln/Gln + Arg / Gln vs. Arg/Arg, OR = 1.49, 95%CI: 1.21-1.83, P <0.001). Compared with Arg-allele, Gln-allele increases the risk of HCC in Chinese population.(OR = 1.33, 95%CI: 1.16-1.54, P <0.001).

Studies included in the meta-analysis for XRCC1 Arg399Gln polymorphism (n=13).

Reference:

Liu J, Zhu QM, Hu HY, Wang S. Association between XRCC1 Arg399Gln polymorphism and susceptibility to hepatocellular carcinoma in Chinese populations: A Meta-analysis. World Chin J Gastroenterol. 2015; 23(15): 2468-2474. DOI: 10.11569/wcjd.v23.i15.2468. http://kns.cnki.net/kns/detail/detail.aspx?FileName=XXHB201515024&DbName=CJFQ2015

Differences:

Firstly, our meta-analysis included more researches, for XRCC1 Arg399Gln polymorphism (n=26). Secondly, Liu et al. did not restrict the analysis to HWE research, so its results might not be stable. In our meta-analysis, XRCC1 Arg399Gln was significantly associated not only with the overall risk of HCC (homozygous model, OR = 1.61, 95% CI: 1.40–1.85, P < 0.05; recessive model, OR = 1.40, 95% CI:1.23–1.59, P < 0.05), but also with the risk of HCC in Chinese patients (homozygous model, OR = 1.78, 95% CI: 1.53–2.08, P < 0.05); recessive model, OR = 1.47, 95% CI: 1.27–1.70, P < 0.05)). Limiting the analysis to studies demonstrating HWE, the results were consistent and robust. Similarly, a significant association between XRCC1 Arg399Gln and HCC risk was found in healthy controls in the general population but not in hospital controls. TSA, FPRP and the combined genotype analysis revealed that XRCC1 Arg399Gln is mainly associated with susceptibility to liver cancer. Therefore, our conclusion is more reliable.

⑦(Xu et al )the XRCC1 Arg280His polymorphism is associated with a higher risk of HCC(HisHis vs ArgArg, OR=1.55, 95%CI:1.10-2.18, P=0.013).

Studies included in the meta-analysis for XRCC1 Arg399Gln polymorphism (n=13).

Reference:

Xu W, Liu S, Li L, Shen ZY, Wu YL. Association between XRCC1 Arg280His polymorphism and risk of hepatocellular carcinoma: a systematic review and meta-analysis. Genet Mol Res. 2015; 14(2): 7122-9. DOI: 10.4238/2015.June.29.5. PMID: 26125922.

Differences:

Firstly, our meta-analysis included more researches, for XRCC1 Arg399Gln polymorph-

ism (n=26). Secondly, we noticed that Xu et al. only based on homozygote model and concluded that Arg280His polymorphism is correlated with HCC. In their research, the other two models are meaningless (Dominant model: His/His+Arg/His vs. Arg/Arg, OR=1.26, 95%CI: 0.95-1.6 8, P=0.115; Recessive model: His/His vs. Arg/His+Arg/Arg, OR=1.30, 95%CI:0.94-1.80, P=0.118). Therefore, we think that the conclusion of Xu et al. is not accurate enough. Secondly, Liu et al. did not restrict the analysis to HWE research, so its results might not be stable. In our meta-analysis, we found that XRCC1 Arg280His polymorphism was not associated with the risk of HCC.
