# Supplementary material for: Associations between three XRCC1 polymorphisms and hepatocellular carcinoma risk: A meta-analysis of case-control studies
Source: PLoS One. 2018 Nov 8;13(11):e0206853. doi: 10.1371/journal.pone.0206853 (PMC6226104; doi:10.1371/journal.pone.0206853)
Supplement: S3 Appendix — (DOC) [file pone.0206853.s008.doc]

Supplemental Information

The detailed search criteria

- 1. “DNA repair pathway” OR “repair gene” OR “repair reaction” OR “repair response” OR “Base Excision Repair/BER” [Mesh]
  2. “X-ray repair cross-complementation group 1” OR “XRCC1” OR “X-ray repair complementing defective repair in Chinese hamster cells 1” [Mesh]
  3. “variation” OR “polymorphism” OR “Single Nucleotide Polymorphism” OR “genetic variability” [Mesh]
  4. “liver cancer” OR “hepatocellular carcinoma” OR “'primary hepatic carcinoma” OR “primary liver cancer” [Mesh]
  5. Strategies 1 through 4
  6. “rs1799782” or “rs25489” or “rs25487”
  7. “Arg194Trp” or “Arg280His” or “Arg399Gln”
  8. Strategy 6 and 7
  9. Strategy 1 and 8

The full data range (year, month) for the search in each database:

PubMed: 1999,1--2018,3

Web of science: 2000,1--2018,03

Cochrane Library: 2002,07--2018,03

the Chinese National Knowledge Infrastructure: 2003,1--2018,03

and the Wanfang standard database: 2003,1--2018,03
